# Copper-catalysed regioselective sulfenylation of indoles with sodium sulfinates

**DOI:** 10.1098/rsos.180170

**Published:** 2018-05-30

**Authors:** Xiaojun Luo, Qiang Liu, Hongxia Zhu, Huoji Chen

**Affiliations:** 1 Integrated Hospital of Traditional Chinese Medicine, Southern Medical University, Shiliugang Road 13th, Guangzhou 510315, People's Republic of China; 2 School of Traditional Chinese Medicine, Southern Medical University, No.1023, South Shatai Road, Baiyun District, Guangzhou 510515, People's Republic of China

**Keywords:** copper catalysis, sulfenylation, indoles, sodium sulfinates

## Abstract

A copper-catalysed sulfenylation of indoles with sodium sulfinates that affords 3-sulfenylindoles in good-to-excellent yields in *N*,*N*-dimethyl formamide (DMF) is described. In the process, DMF serves not only as a solvent but also as a reductant. This transformation is easy to carry out and has mild reaction conditions and good functional group tolerance.

## 1. Introduction

The indole moiety exists in a variety of natural products and synthetic drugs. Therefore, it has always been a hot topic to find a new method for the synthesis of substituted indoles through either construction or modification of indole rings [1,2]. In recent years, 3-sulfenylindoles have attracted more attention because of their broad spectrum of biological and pharmaceutical activities. For example, some 3-sulfenylindoles were investigated as a new class of potent antivirals against the vaccinia virus [3]. Recently, Miller *et al.* reported a 3-sulfenylindole derivative that was a potent inhibitor of small-molecule autotaxin [4]. Furthermore, many 3-sulfenylindole derivatives have been proved to possess other biological activities, including anticancer activity [5,6], anti-HIV-1 activity [7], anti-allergy activity [8] and so on. The indole rings can be directly functionalized with electrophiles via carbon–carbon or carbon-hetero bonds because of their electron-rich nature. In this regard, the sulfenylation of indoles was the most common method for the synthesis of 3-sulfenylindoles. To date, many sulfenylating agents have been used for the sulfenylation of indoles such as thiols [9–13], disulfides [14–18], sulfonyl chlorides [19–21], sulfonyl hydrazides [22–24], *N*-thioimides [25,26] and quinone mono-O,S-acetals [27,28]. However, many of these sulfenylating agents had some disadvantages, such as a foul smell, high cost and being unstable to air and moisture. Furthermore, some methods needed strong oxidants, excess additives or high temperature. Thus, it was desirable to develop a new sulfenylating agent for the synthesis of 3-sulfenylindoles.

**Scheme 1.**
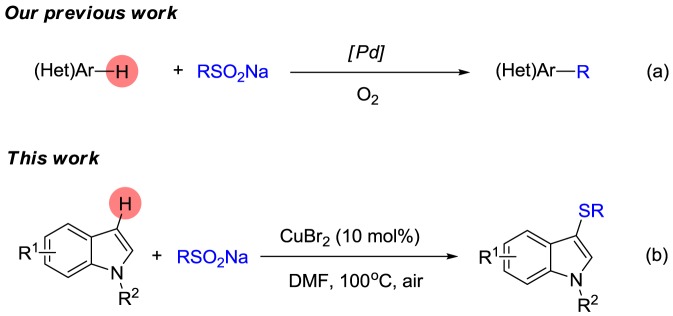
Sulfenylation/arylation of indoles with sodium sulfinates.

Sodium sulfinates can be easily obtained by the reduction of sulfonyl chloride. Because they are inexpensive, stable and easy to handle, they have been widely used as sulfonylating agents [29–32] and arylating agents [33], especially in recent years. In 2014, Deng and co-workers first reported the use of sodium sulfinates as sulfenylating agents for the synthesis of 3-sulfenylindole with dimethyl sulfoxide (DMSO) as the oxidant and diethyl phosphite as the reductant (scheme 1*a*) [32,34–37]. Thereafter, Kuhakarn's group carried out similar work with a I_2_/PPh_3_ reaction system (scheme 1*b*) [38]. Some other groups also developed various reaction systems for realizing the sulfenylation of indoles with sodium sulfinates [39]. However, all these reactions have some disadvantages that involve strong oxidants, excess additives or strong acids.

In 2016, Jiang *et al.* presented excellent work on the copper-catalysed oxysulfenylation reaction of enolates with sodium sulfinates [40]. In the transformation, the sulfur radical was produced in a Cu/*N*,*N*-dimethyl formamide (DMF) reaction system in which DMF not only served as the solvent but also as a reductant. Recently, we reported a new palladium-catalysed direct arylation of heteroarenes with sodium sulfinates (scheme 1*a*) [32]. On the basis of these studies, we wondered whether we could obtain 3-sulfenylindole with sodium sulfinates as sulfenylating agents in a Cu/DMF reaction system. Herein, we present a copper-catalysed sulfenylation of indoles for the synthesis of 3-sulfenylindoles with sodium sulfinates in DMF (scheme 1*b*).

First, indole (**1a**) and sodium phenylsulfinate (**2a**) were chosen as the model substrates for optimization of the conditions, which are summarized in table 1. The desired product **3a** was obtained in a 35% isolated yield in the presence of CuBr_2_ (10 mol %) with PPh_3_ as the reductant (1 equiv) in DMF under air (1 atm) at 100°C for 24 h (table 1, entry 1). We screened different reductants such as Na_2_SO_3_, Na_2_S and (C_2_H_5_O)_2_POH, and found that they were less efficient (table 1, entries 2–4). Without the reductant, the yield was increased up to 82% due to DMF being a reductive solvent [41] (table 1, entry 5). Other additives such as bases (Na_2_CO_3_, NaOH) or acids (HOAc, TsOH) adversely affected the reaction (table 1, entries 6–9). In addition, ligands such as 2,2'-bipyridine, 1,10-phenanthroline and *N*,*N*,*N*′,*N*′-tetramethylethylenediamine were tested; however, they all resulted in decreased yields (table 1, entries 9–12). Other copper salts such as CuCl_2_, Cu(OAc)_2_, Cu(OTf)_2_, CuI, CuBr and CuCl were all tested, but they were less efficient than CuBr_2_ (table 1, entries 13–18). When other solvents (e.g. dichloroethane (DCE), DMSO, dioxane and toluene) were used, no products were formed (table 1, entries 19–22). When the reaction was performed under N_2_, the result was the same as that under air (entry 23). To simplify the operation, we chose to carry out the reaction under air. Thus, the optimized reaction system for this copper-catalysed sulfenylation reaction was: **1a** (0.3 mmol), **2a** (0.4 mmol), CuBr_2_ (10 mol%) in DMF (2 ml) at 100°C under air for 24 h.

**Table 1.** Optimization of reaction conditions^a^ 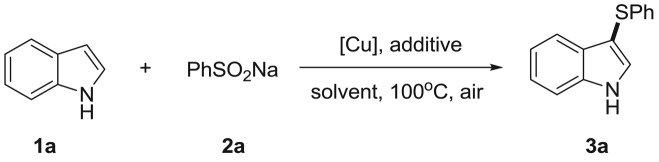
.

entry	[Cu]	additive	solvent	yield (%)
1	CuBr_2_	PPh_3_	DMF	35
2	CuBr_2_	Na_2_SO_3_	DMF	n.d.
3	CuBr_2_	Na_2_S	DMF	n.d.
4	CuBr_2_	(C_2_H_5_O)_2_POH	DMF	41
5	CuBr_2_	—	DMF	82
6	CuBr_2_	Na_2_CO_3_	DMF	65
7	CuBr_2_	NaOH	DMF	54
8	CuBr_2_	HOAc	DMF	67
9	CuBr_2_	TsOH	DMF	52
10^b^	CuBr_2_	—	DMF	64
11^c^	CuBr_2_	—	DMF	67
12^d^	CuBr_2_	—	DMF	51
13	CuCl_2_	—	DMF	63
14	Cu(OAc)_2_	—	DMF	46
15	Cu(OTf)_2_	—	DMF	32
16	CuI	—	DMF	37
17	CuBr	—	DMF	69
18	CuCl	—	DMF	51
19	CuBr_2_	—	DCE	n.d.
20	CuBr_2_	—	DMSO	n.d.
21	CuBr_2_	—	dioxane	n.d.
22	CuBr_2_	—	toluene	n.d.
23^e^	CuBr_2_	—	DMF	80

^a^Reaction conditions: unless otherwise noted, all reactions were performed with **1a** (0.3 mmol), **2a** (0.4 mmol), Cu catalyst (10 mol %), additive (0.3 mmol) and solvent (2 ml), at 100°C under air for 24 h. Isolated yield.

^b^2,2'-Dipyridyl (0.06 mmol) was added.

^c^1,10-Phenanthroline (0.06 mmol) was added.

^d^*N*,*N*,*N*′,*N*′-Tetramethylethylenediamine (0.06 mmol) was added.

^e^Under N_2_ atmosphere.

With the optimized conditions, various sodium arylsulfinates were examined and the results are summarized in table 2. Sodium benzenesulfinates bearing the alkyl substituents at the *para* position smoothly reacted with indole to give the 3-arylthioindoles in good yields (table 2, **3a**–**3c**). A strong electron-donating group such as methoxy was also tested for this reaction (table 2, **3d**). Notably, halo substituents such as fluoro, chloro and bromo were well tolerated and provided the corresponding products in 83%, 90% and 88% yields, respectively (table 2, **3e–3g**). Ortho- and meta-substituted substrates were also applicable to this reaction (table 2, **3 h–3j**).

**Table 2.** Substrate scope of various sodium sulfinates.^a^

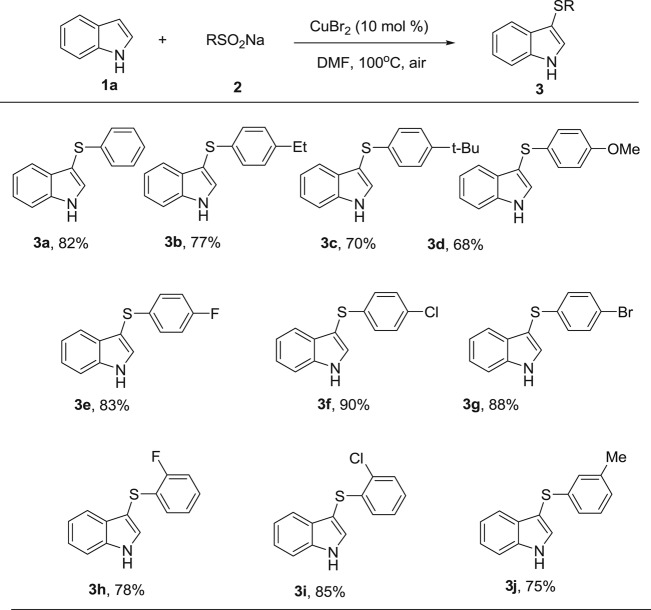

^a^Reaction conditions: **1a** (0.3 mmol), **2** (0.4 mmol), CuBr_2_ (10 mol%), DMF (2 ml), under air at 100^°^C for 24 h. Isolated yields.

Next, various substituted indoles were examined and the results are illustrated in table 3. The reaction of *N*-methylindoles proceeded smoothly and the corresponding product was obtained in an 87% yield (table 3, **3k**). For methyl-substituted indoles at C2 and C5, the yields were 66% and 85%, respectively (table 3, **3l, 3p**). Various functional groups such as –OMe, –F, –Cl, –Br, –COOMe and NO_2_ were tolerated, and provided the products in good–to-excellent yields (table 3, **3m–3u**). In general, electron-donating groups were good for the transformation. The polysubstituted indoles were also applicable to the reaction system (table 3, **3v–3w**).

**Table 3.** Substrate scope of various indoles.^a^

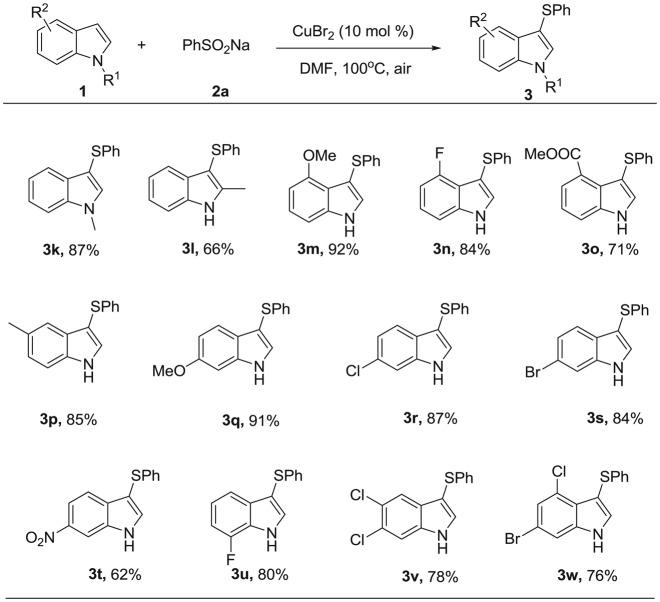

^a^Reaction conditions: **1** (0.3 mmol), **2a** (0.4 mmol), CuBr_2_ (10 mol%), DMF (2 ml), under air at 100°C for 24 h. Isolated yields.

**Scheme 2.**
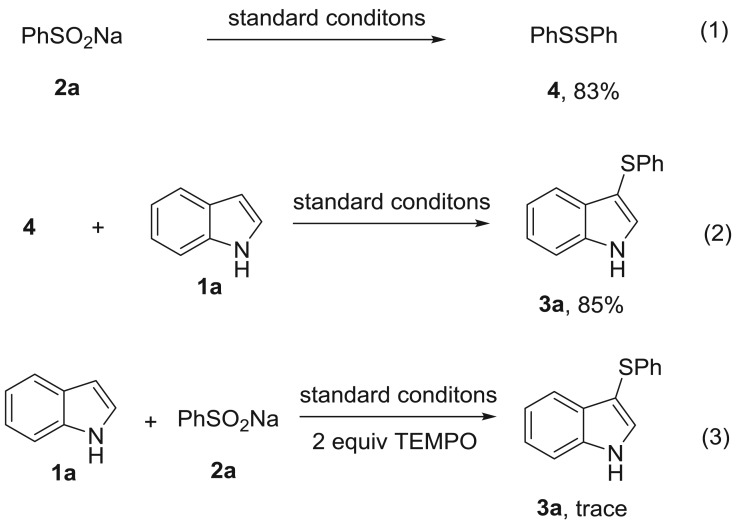
Control experiments.

**Scheme 3.**
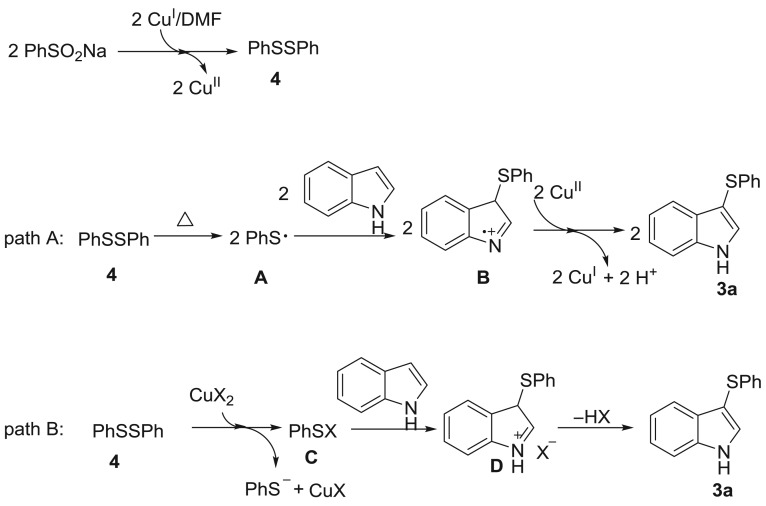
Possible reaction mechanism.

To gain some insight into the possible reaction mechanisms, several control experiments were then carried out. Sodium phenylsulfinate could be converted to 1,2-diphenyldisulfane **4** with an 83% yield in the absence of indole under standard conditions (scheme 2, equation 1). 1,2-Diphenyldisulfane **4** realized the sulfenylation of indole **1a** and provided product **3a** with an 85% yield (scheme 2, equation 2). These two experiments indicated that 1,2-diphenyldisulfane **4** may be a key intermediate in this reaction. In addition, the radical scavenger 2,2,6,6-tetramethylpiperidin-1-yl)oxyl (TEMPO) could suppress the reaction (scheme 2, equation 3). Based on the control experiments and previous work [9,39–43], plausible reaction mechanisms are proposed in scheme 3. Initially, 1,2-diphenyldisulfane **4** was generated from sodium phenylsulfinate in the [Cu]/DMF reaction system [42–44]. Subsequently, the thio radical **A** was produced via direct radical cracking and then radical addition to indole resulted in the formation of intermediate **B**. Finally, a single-electron oxidation followed by aromatization gave the product **3a** (path A) [9]. However, another reaction mechanism may also be possible. Firstly, 1,2-diphenyldisulfane **4** reacts with CuX_2_ to form an electrophilic species intermediate **C**. Then the final product **3a** was formed by electrophilic addition/deprotonation of intermediate **C** (path B) [9].

In conclusion, we have developed a new copper-catalysed method for the synthesis of 3-sulfenylindoles from indoles and sodium sulfinates. Under the optimal reaction conditions, a variety of indoles could be transformed to corresponding 3-sulfenylindoles with good yields and excellent functional group tolerance. Moreover, DMF was used not only as a solvent but also as a reductant in this process. Further study on this topic is currently underway in our laboratory.

## Supplementary Material

Supporting Information

## Data accessibility

This article does not contain any additional data.
