# Supplementary material for: Copper-catalysed regioselective sulfenylation of indoles with sodium sulfinates
Source: R Soc Open Sci. 2018 May 30;5(5):180170. doi: 10.1098/rsos.180170 (PMC5990840; doi:10.1098/rsos.180170)
Supplement: Supporting Information [file rsos180170supp1.doc]

**Supporting Information**

**Copper-catalyzed regioselective sulfenylation of indoles with sodium sulfinates**

Xiaojun Luo ^a^ , Qiang Liu, Hongxia Zhu*^a^ and Huoji Chen*^b^

*^a^Cancer Center，Integrated Hospital of Traditional Chinese Medicine，Southern Medical University. Guangzhou，P. R. China. E-mail:* *gzzhx2012@163.com*

*^b^School of Traditional Chinese Medicine, Southern Medical University, 1023 South Shatai Road, Baiyun District, Guangzhou 510515, P. R. China. E-mail: chenhuoji2005@126.com.*

**Table of Contents**

General Information......................................................................................................................................[2]

Experimental Procedure for Compounds **3**....................................................................................................[2]

Characterization Data for All Products **3**.......................................................................................................[2]

References…………................………………………………………………………….………….……....[8]

NMR Spectra for All Compounds **3**…………….….………………...…………...…..............…...……[9]

**General Information**

^1^H and ^13^C NMR spectra were recorded on BRUKER DRX-400 spectrometer using CDCl_3_ as solvent and TMS as an internal standard. IR spectra were obtained either as potassium bromide pellets or as liquid films between two potassium bromide pellets with a spectrometer. GC-MS was obtained using electron ionization. HRMS was obtained with a LCMS-IT-TOF mass spectrometer. TLC was performed by using commercially prepared 100-400 mesh silica gel plates, and visualization was effected at 254 nm.

**Experimental Procedure for Compounds 3**

The mixture of indoles **1** (0.3 mmol), sodium sulfinates **2** (0.4 mmol), CuBr_2_ (10 mol %) in DMF (2 mL) was stirred at 100 °C under air for 24 h. At ambient temperature, the reaction mixture was diluted with H_2_O (15 mL) and extracted with EtOAc (3 × 15 mL). The organic extracts were dried over MgSO_4_. After filtration and evaporation of the solvents under reduced pressure, the crude product was purified by column chromatography on silica gel to afford desired product.

**Characterization Data for All Products** **3**

**3-(Phenylthio)-1*H*-indole (3a)^1^**

White solid (55.4 mg, 82%); m.p. = 149-150 ^o^C.  ^1^H NMR (400 MHz, CDCl3) δ 8.35 (s, 1H), 7.63 (d, J = 7.9 Hz, 1H), 7.48 – 7.41 (m, 2H), 7.27 (t, J = 8.3 Hz, 1H), 7.21 – 7.04 (m, 6H). ^13^C NMR (100 MHz, CDCl_3_) δ 139.2, 136.5, 130.6, 129.1, 128.7, 125.9, 124.8, 123.0, 120.9, 119.6, 111.6, 102.8. IR (KBr): 3409, 3024, 1652, 1601, 1452, 1402, 741 cm^-1^. MS (EI, 70 eV) *m/z*: 77, 121, 148, 193, 225.

**3-((4-Ethylphenyl)thio)-1*H*-indole (3b)^1^**

White solid (58.4 mg, 77%); m.p. = 115-116 ^o^C. ^1^H NMR (400 MHz, CDCl_3_) δ 8.26 (s, 1H), 7.61 (d, *J* = 7.9 Hz, 1H), 7.40 – 7.34 (m, 2H), 7.25 – 7.19 (m, 1H), 7.13 (t, *J* = 7.5 Hz, 1H), 7.06 – 7.02 (m, 2H), 6.97 (d, *J* = 8.2 Hz, 2H), 2.52 (q, *J* = 7.6 Hz, 2H), 1.14 (t, *J* = 7.6 Hz, 3H). ^13^C NMR (100 MHz, CDCl_3_) δ 141.1, 136.4, 135.7, 130.5, 129.1, 128.3, 126.2, 122.9, 120.8, 119.6, 111.5, 103.3, 28.2, 15.5. IR (KBr): 3405, 3050, 2937, 1634, 1567, 1452, 743 cm^-1^. MS (EI, 70 eV) *m/z*: 77, 121, 148, 238, 253.

**3-((4-(*tert*-Butyl)phenyl)thio)-1*H*-indole (3c)****^1^**

White solid (59.0 mg, 70%); m.p. = 135-136 ^o^C. ^1^H NMR (400 MHz, CDCl_3_) δ 8.29 (s, 1H), 7.57 (d, *J* = 7.9 Hz, 1H), 7.38 – 7.33(m, 2H), 7.20 – 7.14 (m, 2H), 7.13 – 7.05 (m, 2H), 6.97 (d, *J* = 8.3 Hz, 2H), 1.16 (s, 9H). ^13^C NMR (100 MHz, CDCl_3_) δ 147.9, 136.4, 135.7, 130.5, 129.3, 125.8, 125.7, 123.0, 120.8, 119.7, 111.5, 103.4, 34.3, 31.3. IR (KBr): 3395, 3037, 2934, 1655, 1557, 1461, 746 cm^-1^. MS (EI, 70 eV) *m/z*: 77, 119, 148, 250, 281.

**3-((4-Methoxyphenyl)thio)-1*H*-indole (3d)^1^**

White solid (52.0 mg, 68%). m.p. = 112-114 ^o^C. ^1^H NMR (400 MHz, CDCl_3_) δ 8.28 (s, 1H), 7.62 (d, *J* = 7.9 Hz, 1H), 7.42 – 7.32 (m, 2H), 7.22 (t, *J* = 7.6 Hz, 1H), 7.14 – 7.08 (m, 2H), 6.72 (d, *J* = 8.6 Hz, 2H), 3.70 (s, 3H). ^13^C NMR (100 MHz, CDCl_3_) δ 157.8, 136.4, 130.0, 129.5, 129.0, 128.5, 122.9, 120.7, 119.6, 114.5, 111.5, 104.5, 55.3. IR (KBr): 3382, 3045, 2960, 1647, 1581, 1451, 743 cm^-1^. MS (EI, 70 eV) m/z: 77, 148, 212, 240, 255.

**3-((4-Fluorophenyl)thio)-1*H*-indole (3e)^1^**

Whtie solid (60.5 mg, 83%). m.p. = 133-134 ^o^C. ^1^H NMR (400 MHz, CDCl_3_) δ 8.30 (s, 1H), 7.58 (d, *J* = 7.9 Hz, 1H), 7.43 – 7.35 (m, 2H), 7.24 (t, *J* = 7.6 Hz, 1H), 7.15 (t, *J* = 7.5 Hz, 1H), 7.11 – 7.02 (m, 2H), 6.84 (t, *J* = 8.1 Hz, 2H). ^13^C NMR (100 MHz, CDCl_3_) δ 160.9 (d, *J* = 243 Hz), 136.5, 134.0 (d, *J* = 3 Hz), 130.5, 128.8, 127.9 (d, *J* = 8 Hz), 123.1, 120.9, 119.5, 115.7 (d, *J* = 22 Hz), 111.6, 103.3. IR (KBr): 3401, 3027, 1632, 1583, 1524, 1443, 745 cm^-1^. MS (EI, 70 eV) m/z: 77, 121, 211, 243.

**3-((4-Chlorophenyl)thio)-1*H*-indole (3f)^1^**

White solid (69.9 mg, 90%); m.p. = 127-128 ^o^C. ^1^H NMR (400 MHz, CDCl_3_) δ 8.36 (s, 1H), 7.56 (d, *J* = 7.9 Hz, 1H), 7.44 – 7.40 (m, 2H), 7.28 – 7.22 (m, 1H), 7.16 (t, *J* = 7.5 Hz, 1H), 7.10 (d, *J* = 8.6 Hz, 2H), 7.00 (d, *J* = 8.5 Hz, 2H). ^13^C NMR (100 MHz, CDCl_3_) δ 137.8, 136.5, 130.7, 130.5, 128.8, 128.7, 127.1, 123.2, 121.0, 119.5, 111.7, 102.4. IR (KBr): 3393, 3025, 1655, 1546, 1476, 1407, 745 cm^-1^. MS (EI, 70 eV) *m/z*: 77, 121, 148, 165, 259.

**3-((4-Bromophenyl)thio)-1*H*-indole (3g)^1^**

White solid (80.0 mg, 88%); m.p. = 141-142 ^o^C. ^1^H NMR (400 MHz, CDCl_3_) δ 8.39 (s, 1H), 7.74 (s, 1H), 7.43 (s, 1H), 7.32 (d, *J* = 8.6 Hz, 1H), 7.26 (d, *J* = 8.6 Hz, 1H), 7.16 (t, *J* = 7.6 Hz, 2H), 7.07 (d, *J* = 7.3 Hz, 3H). ^13^C NMR (101 MHz, CDCl_3_) δ 138.7, 135.1, 131.8, 130.9, 128.8, 126.1, 125.9, 125.0, 122.2, 114.4, 113.1, 102.7. IR (KBr): 3402, 3045, 1636, 1545, 1446, 1403, 743 cm^-1^. MS (EI, 70 eV) *m/z*: 77, 111, 148, 203, 271, 303.

**3-((2-Fluorophenyl)thio)-1*H*-indole (3h)^2^**

White solid (56.9 mg, 78%); m.p. = 144-145 ^o^C. ^1^H NMR (400 MHz, CDCl_3_) δ 8.42 (s, 1H), 7.65 (d, *J* = 7.9 Hz, 1H), 7.50 (s, 1H), 7.45 (d, *J* = 8.1 Hz, 1H), 7.30 (t, *J* = 7.6 Hz, 1H), 7.20 (t, *J* = 7.5 Hz, 1H), 7.04 (d, *J* = 7.0 Hz, 2H), 6.88 – 6.79 (m, 2H). ^13^C NMR (100 MHz, CDCl_3_) δ 159.1 (d, *J* = 242 Hz), 136.5, 131.2, 129.1, 128.1 (d, *J* =3 Hz), 126.4 (d, *J* =17 Hz), 126.3 (d, *J* = 7 Hz), 124.3 (d, *J* = 3 Hz), 123.1, 121.0, 119.5, 115.1 (d, *J* = 21 Hz), 111.7, 100.7. IR (KBr): 3401, 3036, 1624, 1581, 1438, 1411, 746 cm^-1^. MS (EI, 70 eV) *m/z*: 77, 121, 148, 183, 243.

**3-((2-Chlorophenyl)thio)-1*H*-indole (3i)****^3^**

White solid (66.0 mg, 85%); m.p. = 136-137 ^o^C. ^1^H NMR (400 MHz, CDCl_3_) δ 8.43 (s, 1H), 7.62 (d, *J* = 1.9 Hz, 1H), 7.46 (d, *J* = 2.6 Hz, 1H), 7.33 (d, *J* = 8.6 Hz, 1H), 7.24 – 7.17 (m, 3H), 7.14 – 7.10 (m, 3H). ^13^C NMR (100 MHz, CDCl_3_) δ 138.7, 134.8, 132.0, 130.3, 128.8, 126.8, 125.9, 125.0, 123.5, 119.0, 112.7, 102.7. IR (KBr): 3389, 1654, 1554, 1496, 1471, 1412, 747 cm^-1^. MS (EI, 70 eV) *m/z*: 77, 155, 223, 259.

**3-(*m*-Tolylthio)-1*H*-indole (3j)^4^**

White solid (53.8 mg, 75%); m.p. = 124-125 ^o^C. ^1^H NMR (400 MHz, CDCl_3_) δ 8.27 (s, 1H), 7.60 (d, *J* = 7.9 Hz, 1H), 7.41 – 7.35 (m, 2H), 7.26 – 7.21 (m, 1H), 7.14 (t, *J* = 7.5 Hz, 1H), 7.02 (d, *J* = 8.1 Hz, 2H), 6.95 (d, *J* = 8.1 Hz, 2H), 2.23 (s, 3H). ^13^C NMR (100 MHz, CDCl_3_) δ 136.4, 135.4, 134.6, 130.4, 129.5, 129.1, 126.3, 122.9, 122.7, 120.8, 120.3, 119.6, 111.5, 103.4, 20.8. IR (KBr): 3401, 3034, 2925, 1767, 1628, 1582, 1443, 1221, 743 cm^-1^. MS (EI, 70 eV) *m/z*: 77, 121, 148, 207, 239.

**1-Methyl-3-(phenylthio)-1*H*-indole (3k)^1^**

White solid (62.4 mg, 87%); m.p. = 83-85 ^o^C. ^1^H NMR (400 MHz, CDCl_3_) δ 7.59 (d, *J* = 7.8 Hz, 1H), 7.34 (d, *J* = 8.1 Hz, 1H), 7.25 (d, *J* = 10.0 Hz, 2H), 7.16 – 7.05 (m, 5H), 7.00 (t, *J* = 5.6 Hz, 1H), 3.76 (s, 3H). ^13^C NMR (101 MHz, CDCl_3_) δ 139.6, 137.5, 135.0, 129.8, 128.6, 125.7, 124.6, 122.5, 120.5, 119.7, 109.7, 100.5, 33.0. IR (KBr): 3031, 1613, 1505, 1451, 1376, 744 cm^-1^. MS (EI, 70 eV) *m/z*: 77, 119, 162, 207, 239.

**2-Methyl-3-(phenylthio)-1*H*-indole (3l)^1^**

White solid (47.3 mg, 66%); m.p. = 113-115 ^o^C. ^1^H NMR (400 MHz, CDCl_3_) δ 8.10 (s, 1H), 7.53 (d, *J* = 7.7 Hz, 1H), 7.28 (d, *J* = 8.0 Hz, 1H), 7.21 – 7.08 (m, 4H), 7.05 – 7.00 (m, 3H), 2.45 (s, 3H). ^13^C NMR (100 MHz, CDCl_3_) δ 141.1, 139.3, 135.4, 130.3, 128.7, 125.5, 124.5, 122.1, 120.7, 118.9, 110.6, 99.3, 12.1. IR (KBr): 3397, 2922, 1723, 1584, 1443, 748 cm^-1^. MS (EI, 70 eV) m/z: 77, 118, 162, 207, 239.

**4-Methoxy-3-(phenylthio)-1*H*-indole (3m)**

White solid (70.4 mg, 92%); m.p. = 79-80 ^o^C. ^1^H NMR (400 MHz, CDCl_3_) δ 8.33 (s, 1H), 7.23 – 7.09 (m, 6H), 7.03 (s, 1H), 6.96 (d, *J* = 8.1 Hz, 1H), 6.51 (d, *J* = 7.8 Hz, 1H), 3.66 (s, 3H). ^13^C NMR (100 MHz, CDCl_3_) δ 154.6, 140.6, 138.4, 129.5, 128.4, 126.4, 124.6, 123.9, 118.6, 104.8, 102.4, 101.5, 55.5. IR (KBr): 3411, 3032, 1657, 1603, 1561, 1447, 1280, 745 cm^-1^. HRMS (ESI) m/z: calcd for C_15_H_13_NNaOS [M+Na]^+^, 278.0610; found, 278.0614.

**4-Fluoro-3-(phenylthio)-1*H*-indole (3n)****^2^**

Yellow solid (61.2 mg, 84%); m.p. = 136-137 ^o^C. ^1^H NMR (400 MHz, CDCl_3_) δ 8.47 (s, 1H), 7.38 (d, *J* = 2.5 Hz, 1H), 7.21 – 7.10 (m, 7H), 6.85 – 6.76 (m, 1H). ^13^C NMR (100 MHz, CDCl_3_) δ 156.9 (d, *J* = 249 Hz), 139.6, 139.3(d, *J* = 10 Hz), 131.0, 128.7, 126.3, 125.0, 123.6 (d, *J* = 7 Hz), 117.7 (d, *J* = 18 Hz), 107.7 (d, *J* = 4 Hz), 106.4 (d, *J* = 19 Hz), 101.3. IR (KBr): 3395, 3023, 1655, 1623, 1437, 1402, 741 cm^-1^. MS (EI, 70 eV) m/z: 77, 95, 122, 166, 211, 243.

**Methyl 3-(phenylthio)-1*H*-indole-4-carboxylate (3o)^5^**

White solid (60.3 mg, 71%); m.p. = 122-123 ^o^C ^1^H NMR (400 MHz, CDCl_3_) δ 9.26 (s, 1H), 7.44 (dd, *J* = 7.8, 3.1 Hz, 2H), 7.35 (s, 1H), 7.20 (dd, *J* = 14.4, 6.8 Hz, 1H), 7.14 – 7.08 (m, 2H), 7.04 – 6.98 (m, 3H), 3.58 (s, 3H). ^13^C NMR (100 MHz, CDCl_3_) δ 169.6, 140.1, 137.5, 133.9, 128.5, 125.5, 125.3, 125.1, 124.6, 122.1, 122.0, 115.2, 101.8, 51.9. IR (KBr): 3407, 3034, 2945, 1682, 1635, 1523, 1424, 765 cm^-1^. MS (EI, 70 eV) m/z: 77, 111, 152, 196, 223, 283.

**5-Methyl-3-(phenylthio)-1*H*-indole (3p)^2^**

White solid (60.9 mg, 85%); m.p. = 134-135 ^o^C. ^1^H NMR (400 MHz, CDCl_3_) δ 8.22 (s, 1H), 7.39 (d, *J* = 7.2 Hz, 2H), 7.28 (d, *J* = 8.2 Hz, 1H), 7.14 (t, *J* = 7.4 Hz, 2H), 7.11 – 7.01 (m, 4H), 2.39 (s, 3H). ^13^C NMR (100 MHz, CDCl_3_) δ 139.5, 134.8, 130.9, 130.4, 129.4, 128.7, 125.6, 124.7, 124.6, 119.1, 111.2, 101.9, 21.4. IR (KBr): 3402, 3031, 2916, 1622, 1537, 1445, 1221, 747 cm^-1^. MS (EI, 70 eV) m/z: 77, 118, 162, 206, 223, 239.

**6-Methoxy-3-(phenylthio)-1*H*-indole (3q)**

Yellow solid (69.6 mg, 91%); m.p. = 116-117 ^o^C. ^1^H NMR (400 MHz, CDCl_3_) δ 8.27 (s, 1H), 7.47 (d, *J* = 8.6 Hz, 1H), 7.36 (s, 1H), 7.21 – 7.11 (m, 4H), 7.06 (t, *J* = 7.0 Hz, 1H), 6.91 (s, 1H), 6.84 (d, *J* = 8.7 Hz, 1H), 3.85 (s, 3H). ^13^C NMR (100 MHz, CDCl_3_) δ 157.2, 139.3, 137.3, 129.4, 128.7, 125.8, 124.7, 123.3, 120.3, 110.8, 102.8, 95.1, 55.7. IR (KBr): 3403, 3034, 2914, 1625, 1537, 1445, 1221, 747 cm^-1^. HRMS (ESI) m/z: calcd for C_15_H_13_NNaOS [M+Na]^+^, 278.0610; found, 278.0609.

**6-Chloro-3-(phenylthio)-1*H*-indole (3r)^1^**

White solid (67.6 mg, 87%); m.p. = 102-103 ^o^C. ^1^H NMR (400 MHz, CDCl_3_) δ 8.54 (s, 1H), 7.48 (d, *J* = 8.4 Hz, 2H), 7.23 (d, *J* = 7.6 Hz, 1H), 7.14 (t, *J* = 7.4 Hz, 2H), 7.09 –7.04 (m, 4H). ^13^C NMR (100 MHz, CDCl_3_) δ 138.6, 133.7, 131.1, 130.5, 128.7, 126.0, 125.0, 122.4, 121.7, 118.3, 117.0, 104.4. IR (KBr): 3398, 3054, 1721, 1634, 1536, 1453, 746 cm^-1^. MS (EI, 70 eV) m/z: 111, 146, 182, 224, 259.

**6-Bromo-3-(phenylthio)-1*H*-indole (3s)**

Yellow solid (76.4 mg, 84%); m.p. = 146-147 ^o^C. ^1^H NMR (400 MHz, CDCl_3_) δ 8.38 (s, 1H), 7.56 (d, *J* = 1.5 Hz, 1H), 7.43 (t, *J* = 5.3 Hz, 2H), 7.26 – 7.22 (m, 1H), 7.18 – 7.13 (m, 2H), 7.08 – 7.03 (m, 3H). ^13^C NMR (100 MHz, CDCl_3_) δ 138.7, 137.2, 131.1, 128.8, 128.0, 126.0, 125.0, 124.3, 121.0, 116.7, 114.5, 103.6. IR (KBr): 3401, 3035, 1636, 1541, 1455, 1403, 756 cm^-1^. HRMS (ESI) m/z: calcd for C_14_H_10_BrNNaS [M+Na]^+^, 325.9610; found, 325.9604.

**6-Nitro-3-(phenylthio)-1*H*-indole (3t)**

Yellow solid (50.2 mg, 62%); m.p. = 148-149 ^o^C. ^1^H NMR (400 MHz, CDCl_3_) δ 9.01 (s, 1H), 8.44 (d, *J* = 1.8 Hz, 1H), 8.06 (dd, *J* = 8.8, 1.9 Hz, 1H), 7.77 (d, *J* = 2.6 Hz, 1H), 7.67 (d, *J* = 8.8 Hz, 1H), 7.22 – 7.16 (m, 2H), 7.10 (dd, *J* = 7.2, 5.2 Hz, 3H). ^13^C NMR (100 MHz, CDCl_3_) δ 144.3, 137.9, 135.7, 135.1, 134.0, 128.9, 126.3, 125.5, 119.9, 116.4, 108.6, 105.1. IR (KBr): 3393, 3025, 1644, 1577, 1435, 1403, 745 cm^-1^. HRMS (ESI) m/z: calcd for C14H10N2NaO2S [M+Na]^+^, 293.0355; found, 293.0358.

**7-Fluoro-3-(phenylthio)-1*H*-indole (3u)**

Yellow solid (58.3 mg, 80%); m.p. = 114-115 ^o^C. ^1^H NMR (400 MHz, CDCl_3_) δ 8.56 (s, 1H), 7.49 (d, *J* = 2.5 Hz, 1H), 7.40 (d, *J* = 7.9 Hz, 1H), 7.20 (t, *J* = 7.5 Hz, 2H), 7.16 – 7.05 (m, 4H), 7.04 – 6.96 (m, 1H). ^13^C NMR (100 MHz, CDCl_3_) δ 149.5 (d, *J* = 244 Hz), 138.7, 132.6 (d, *J* = 4 Hz), 131.1, 128.7, 126.0, 125.0, 124.9 (d, *J* = 14 Hz), 121.2 (d, *J* = 6 Hz), 115.4 (d, *J* = 4 Hz), 107.9 (d, *J* = 16 Hz), 104.1. IR (KBr): 3407, 3045, 1643, 1616, 1542, 1423, 751 cm^-1^. HRMS (ESI) m/z: calcd for C_14_H_11_FNS [M + H]^+^ 244.0591, found 244.0586.

**5,6-Dichloro-3-(phenylthio)-1*H*-indole (3v)**

Yellow solid (68.6 mg, 78%); m.p. = 149-150 ^o^C. ^1^H NMR (400 MHz, CDCl_3_) δ 8.43 (s, 1H), 7.68 (s, 1H), 7.53 (s, 1H), 7.49 (d, *J* = 1.8 Hz, 1H), 7.19 (t, *J* = 7.5 Hz, 2H), 7.09 (t, *J* = 8.3 Hz, 3H). ^13^C NMR (100 MHz, CDCl_3_) δ 138.3, 135.1, 132.5, 128.9, 128.9, 127.2, 126.0, 125.4, 125.2, 120.7, 113.2, 103.2. IR (KBr): 3401, 3026, 1647, 1614, 1535, 1443, 745 cm^-1^. HRMS-ESI (m/z) calcd for C_14_H_9_Cl_2_NNaS [M + Na]^+^ 315.9725, found 315.9730.

**6-Bromo-4-chloro-3-(phenylthio)-1*H*-indole (3w)**

Green solid (76.8 mg, 76%); m.p. = 133-135 ^o^C. ^1^H NMR (400 MHz, CDCl_3_) δ 8.46 (s, 1H), 7.42 (d, *J* = 13.6 Hz, 2H), 7.24 (s, 1H), 7.18 (t, *J* = 7.5 Hz, 2H), 7.11 – 7.06 (m, 3H). ^13^C NMR (100 MHz, CDCl_3_) δ 140.2, 138.3, 133.0, 128.7, 127.7, 126.0, 125.1, 125.0, 124.3, 115.9, 113.5, 103.7. IR (KBr): 3401, 3033, 1652, 1624, 1545, 1511, 1435, 1403, 746 cm^-1^. HRMS-ESI (m/z) calcd for C_14_H_9_BrClNNaS [M + Na]^+^ 359.9220, found 359.9223.

**References**

1. F. Xiao, H. Xie, S. Liu and G.-J. Deng, *Adv. Synth. Cata*l., 2014, **356**, 364.
2. D. Huang, J. Chen, W. Dan, J. Ding, M. Liu and H. Wu, *Adv. Synth. Cata*l., 2012, **354**, 2123.
3. S. Song, Y. Zhang, A. Yeerlan, B. Zhu, J. Liu and N. Jiao, *Angew. Chem. Int. Ed*., 2017, **56**, 2487.
4. Y. Maeda, M. Koyabu, T. Nishimura and S. Uemura, *J. Org. Chem*., 2004, **69**, 7688.
5. H. Zhang, X. Bao, Y. Song, J. Qu and B. Wang, *Tetrahedron*, 2015, **71**, 8885.

**NMR Spectra for All Compounds 3**

**3a**

**
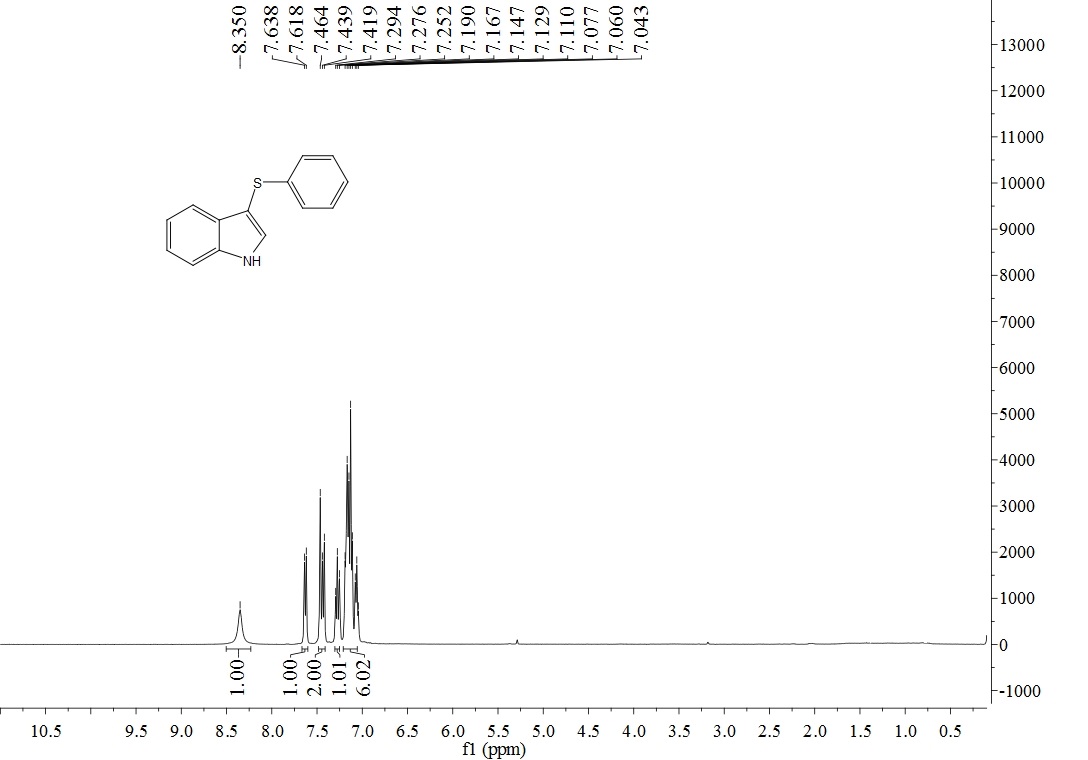
**

**
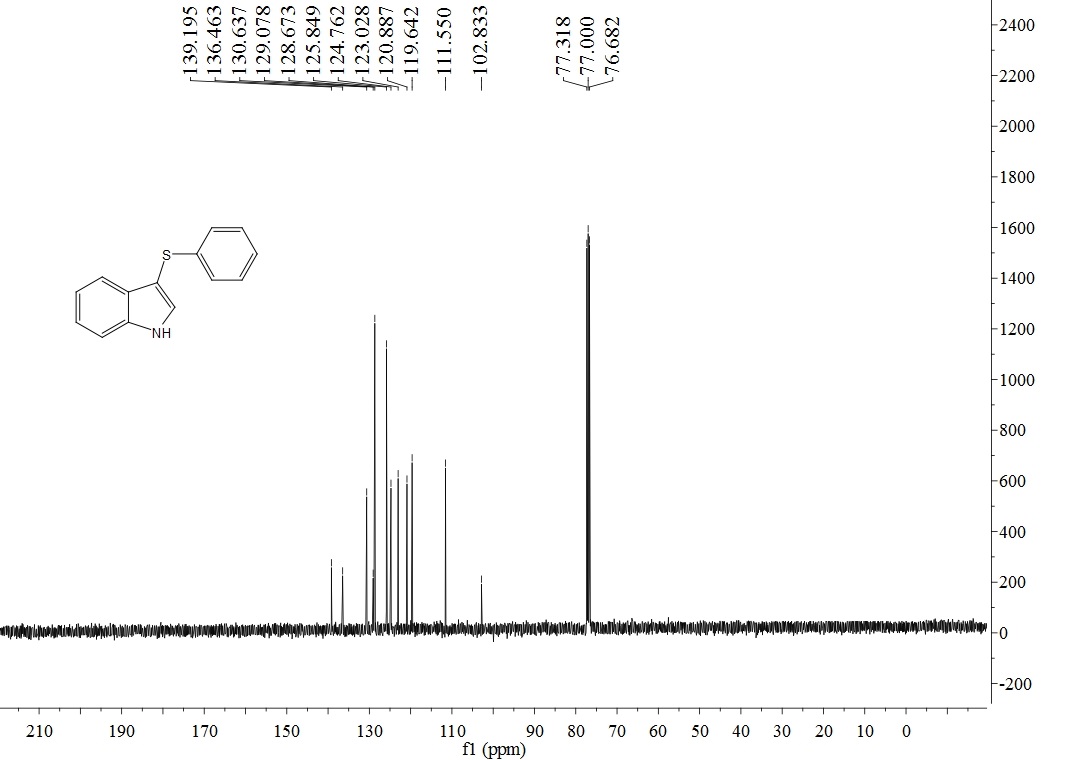
**

**3b**

**
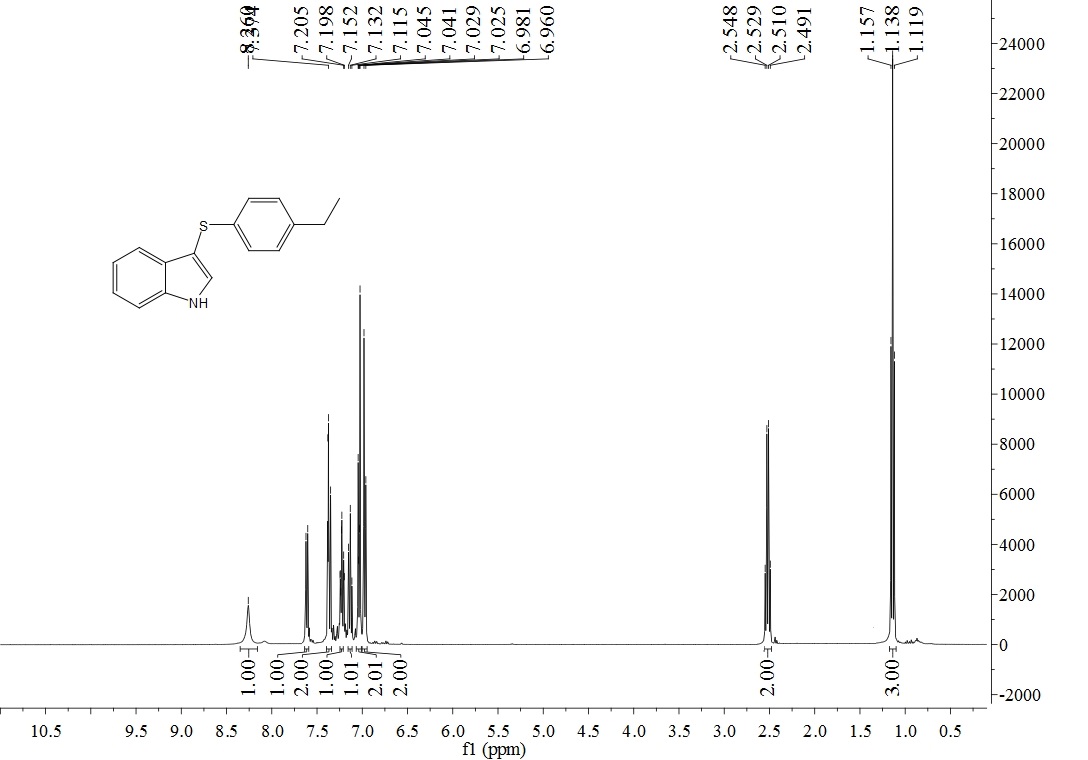
**

**
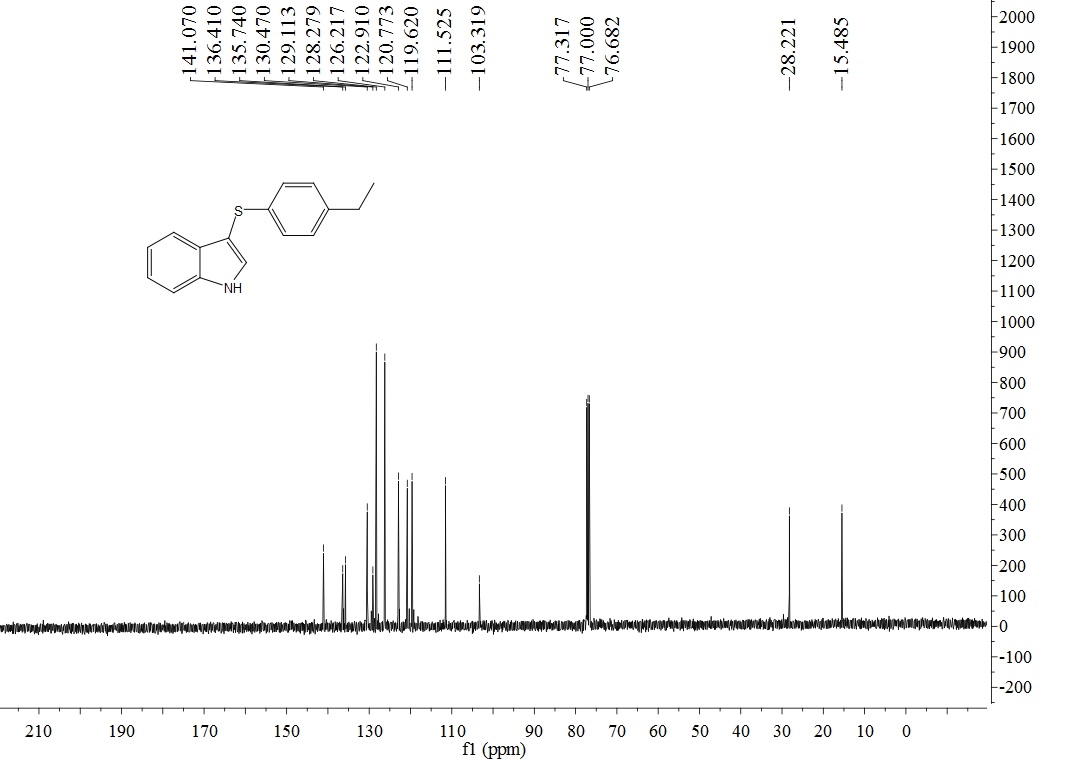
**

**3c**

**
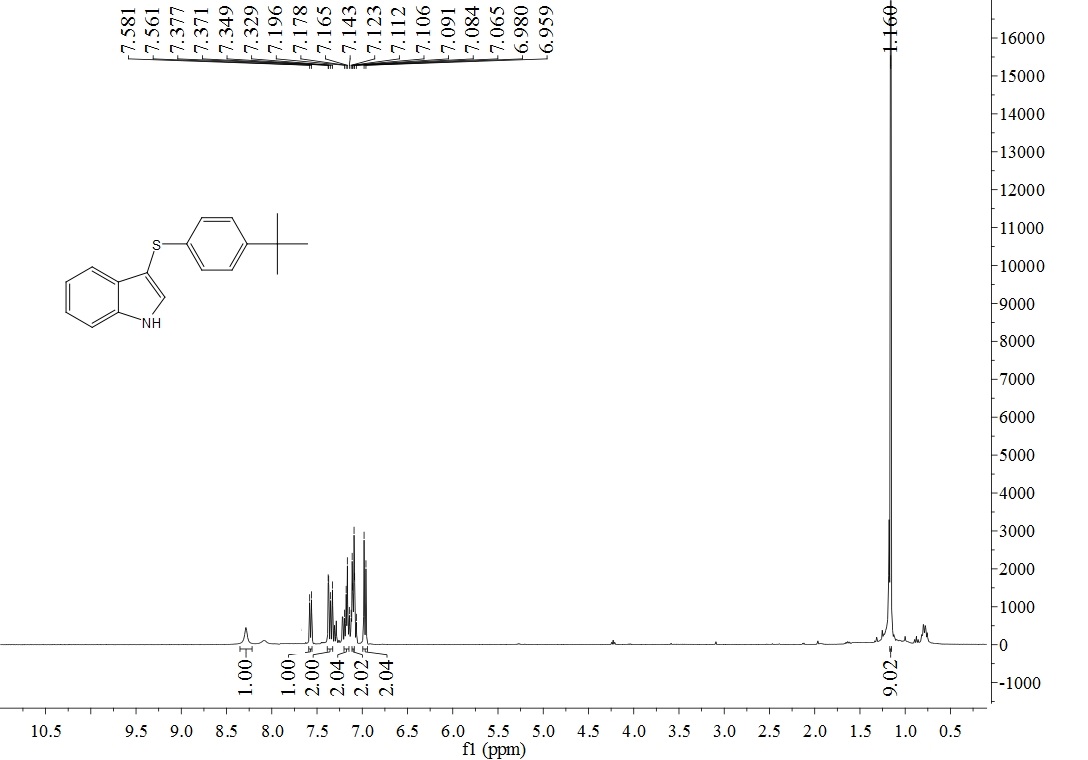
**

**
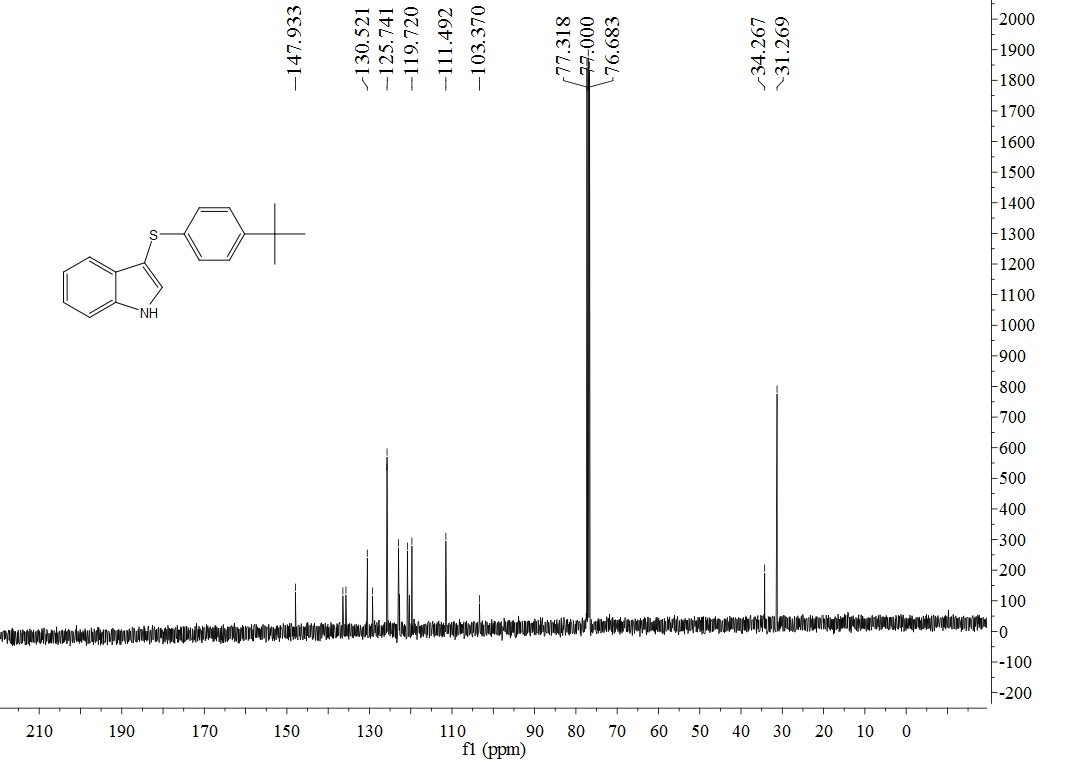
**

**3d**

**
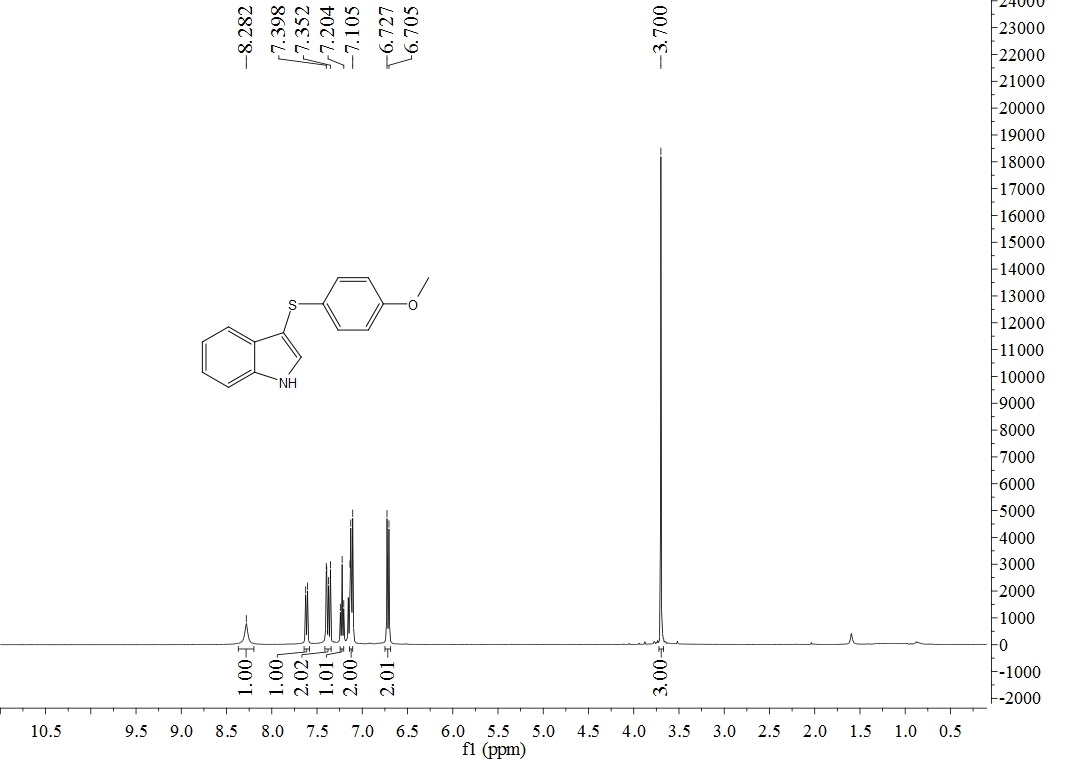
**

**
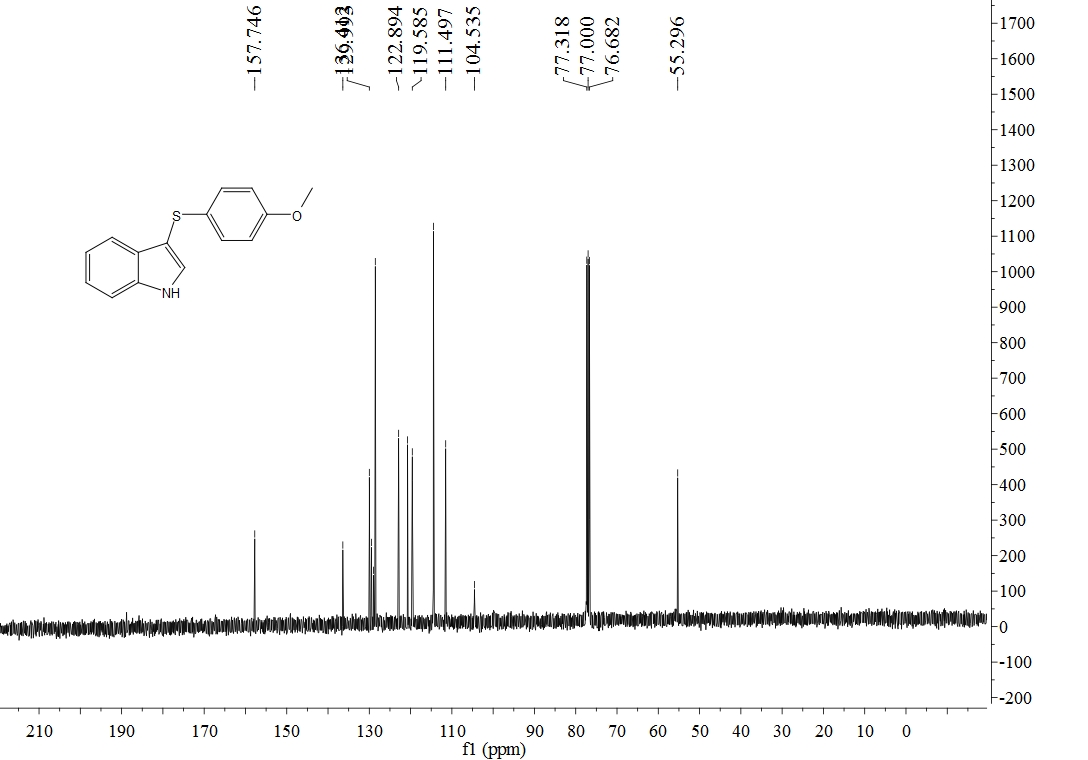
**

**3e**

**
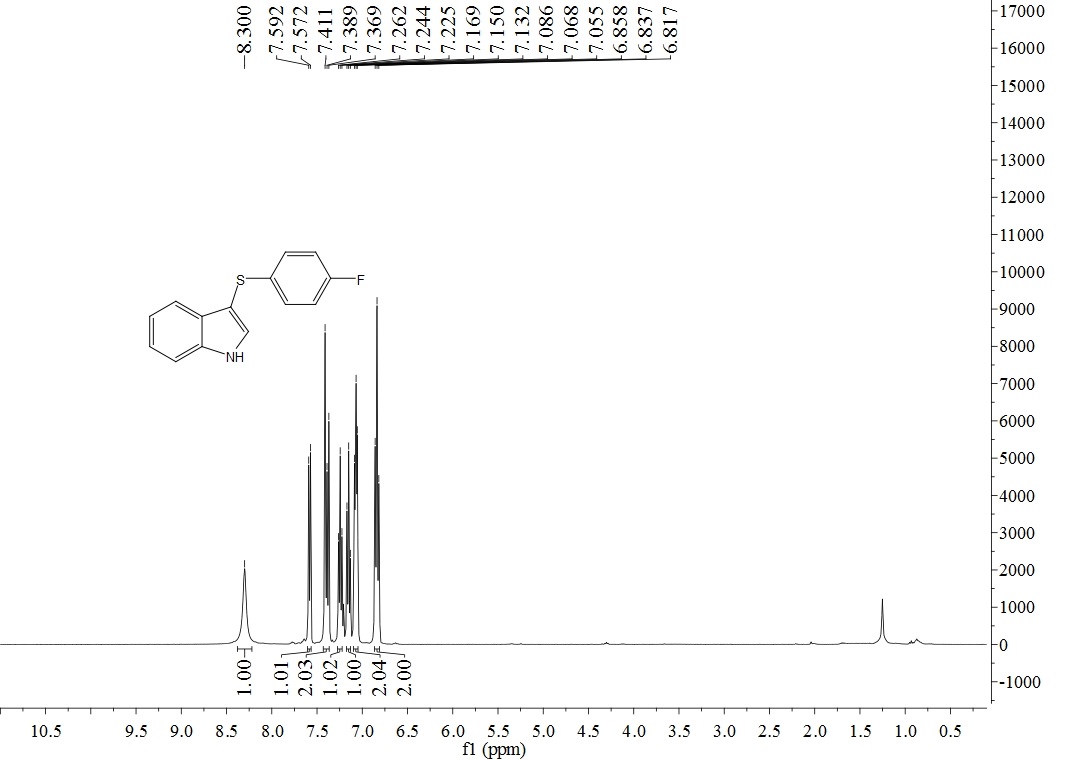
**

**
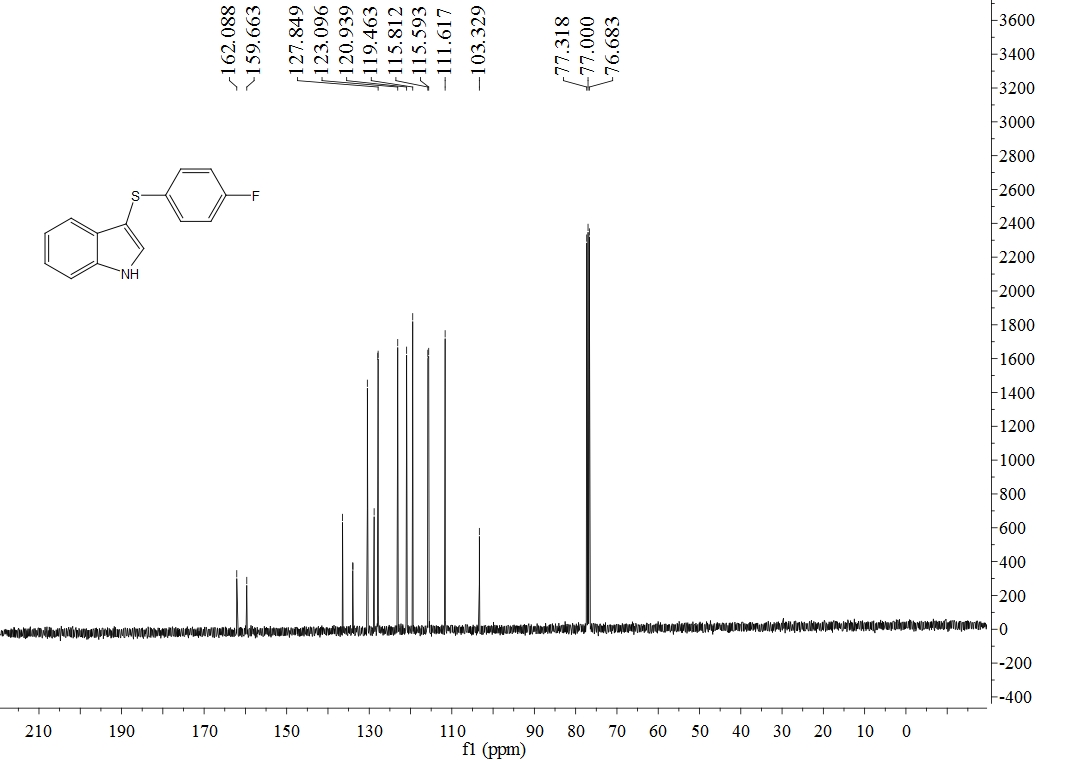
**

**3f**

**
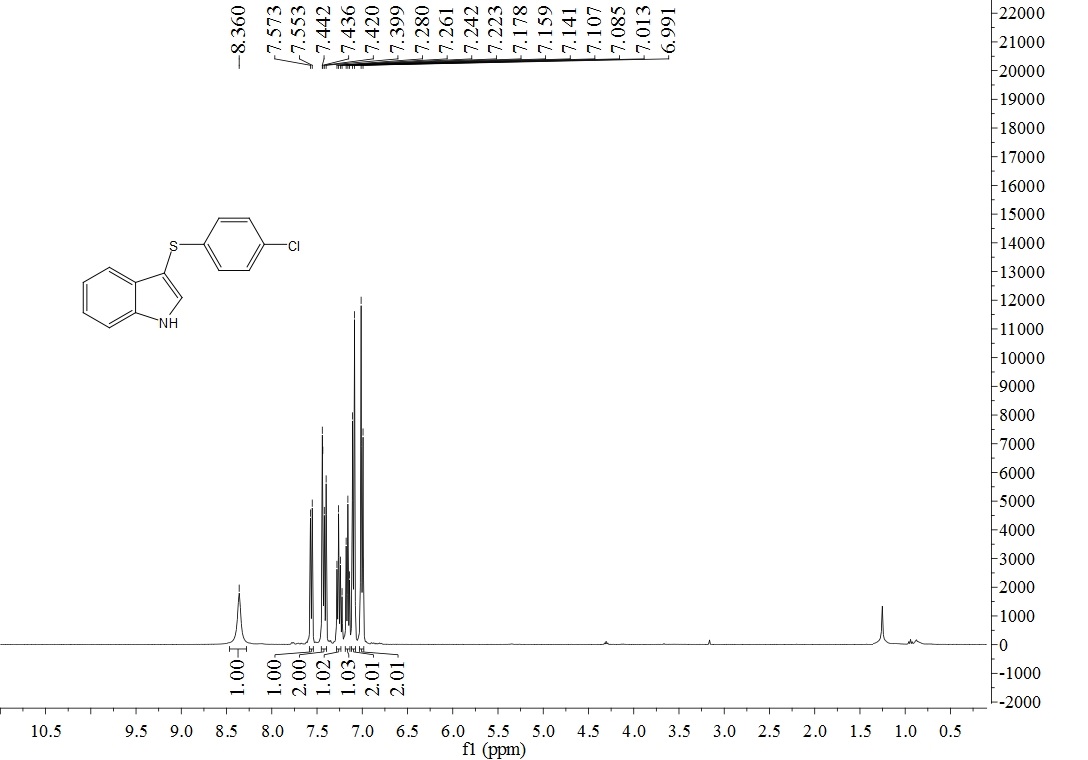
**

**
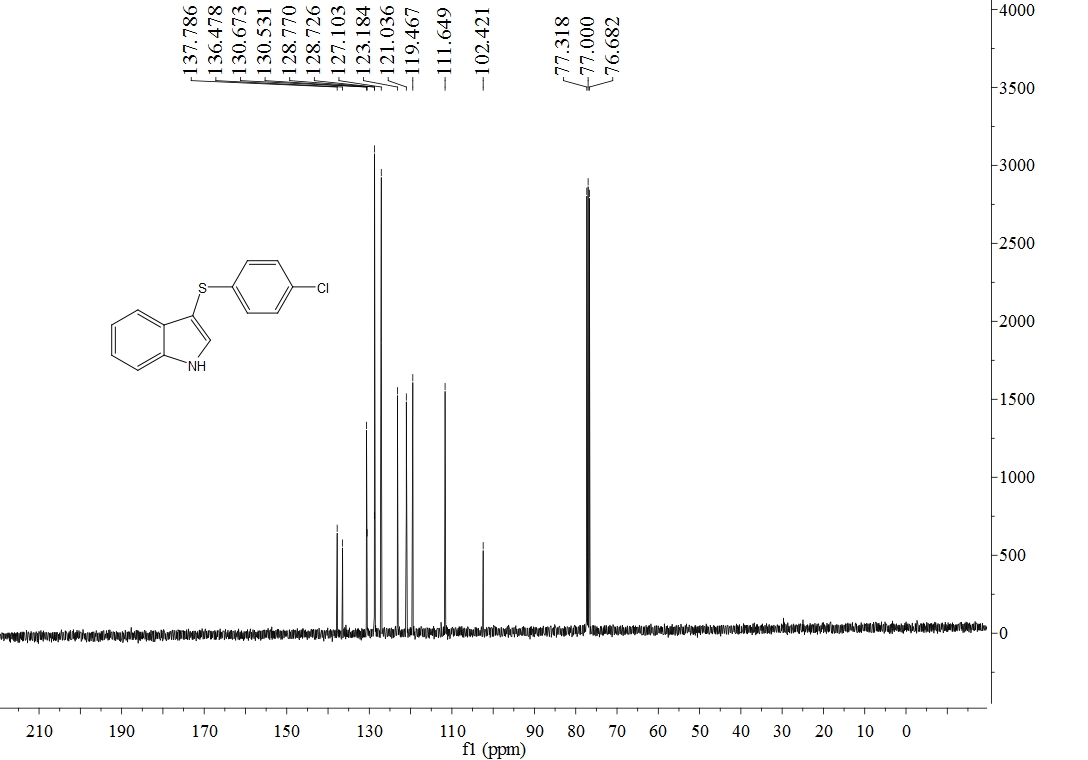
**

**3g**

**
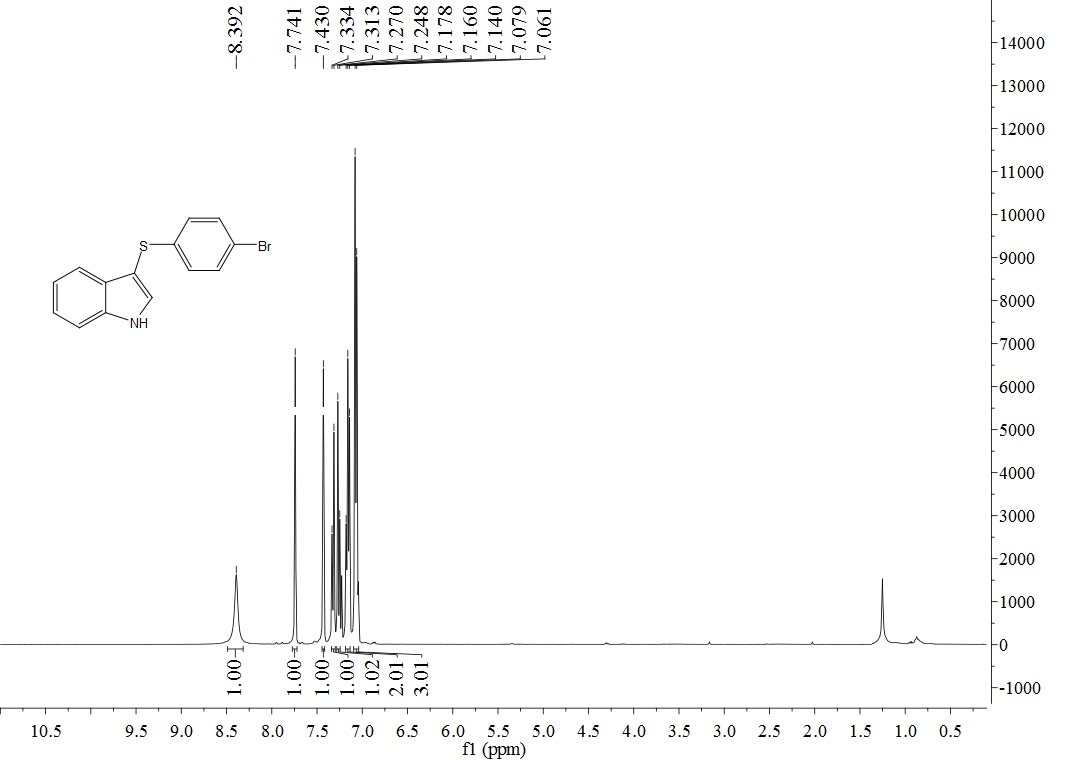
**

**
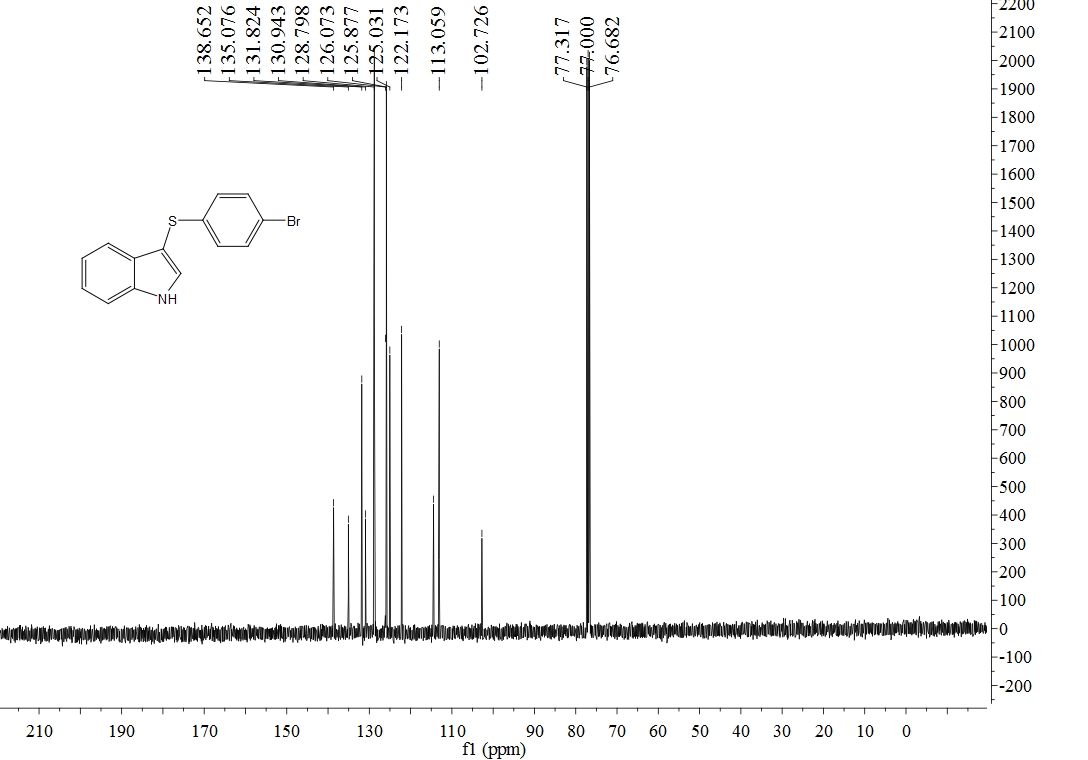
**

**3h**

**
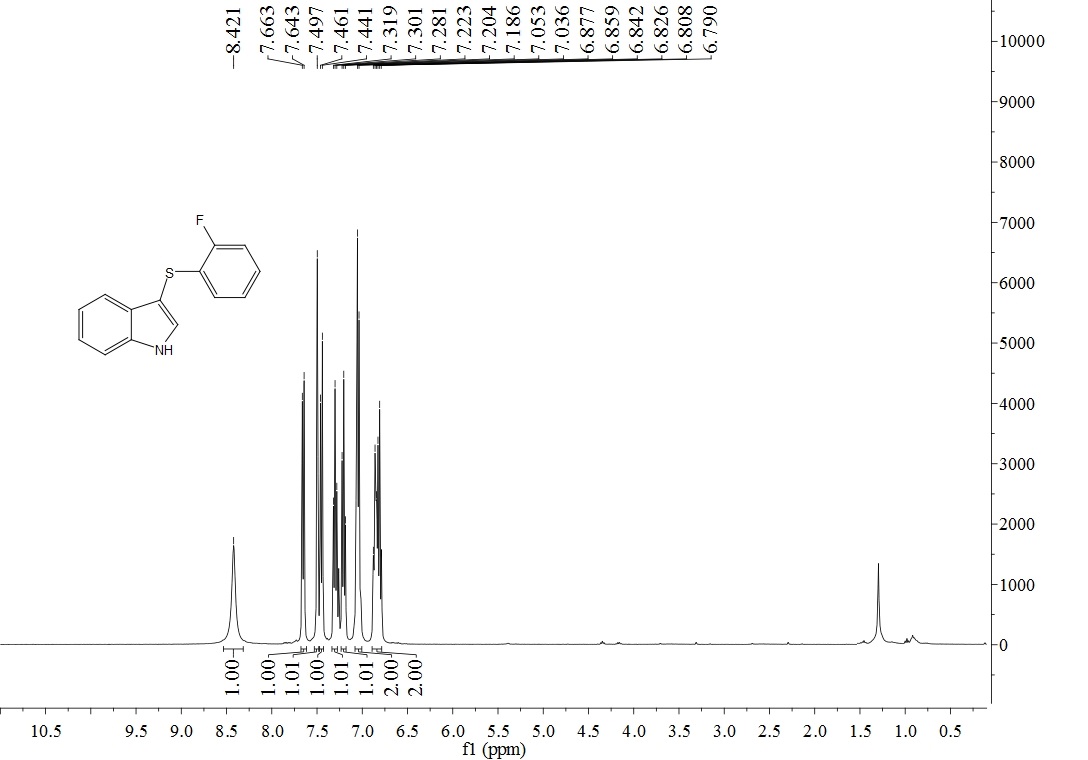
**

**
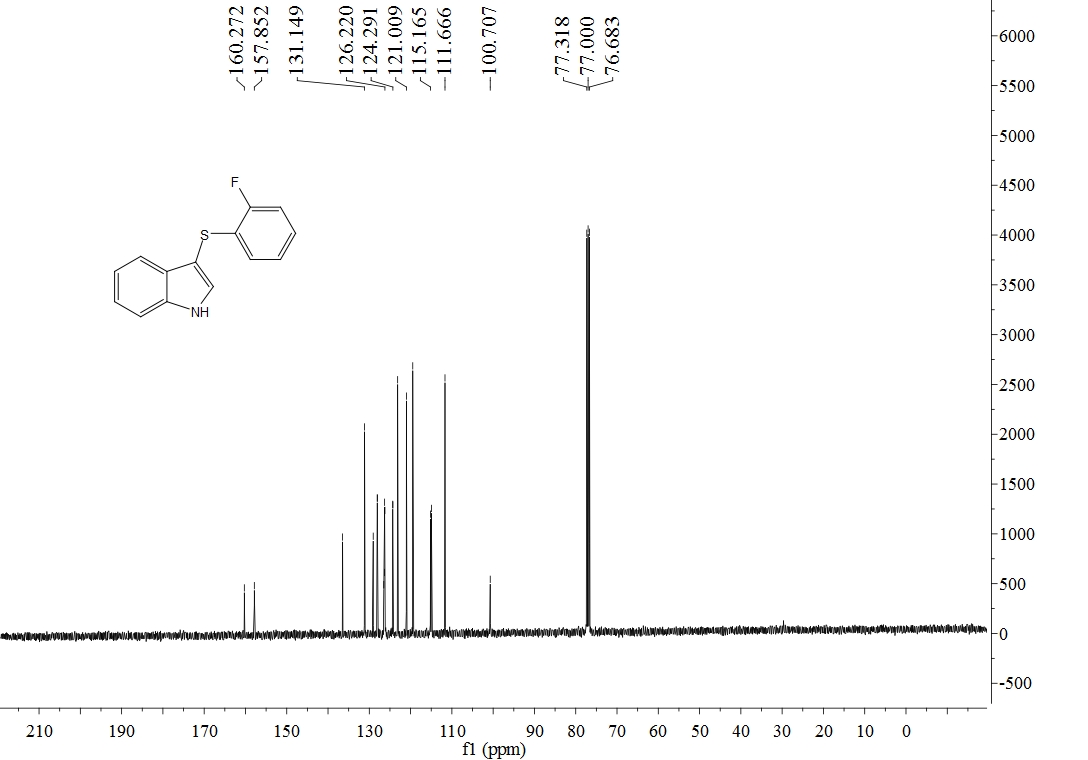
**

**3i**

**
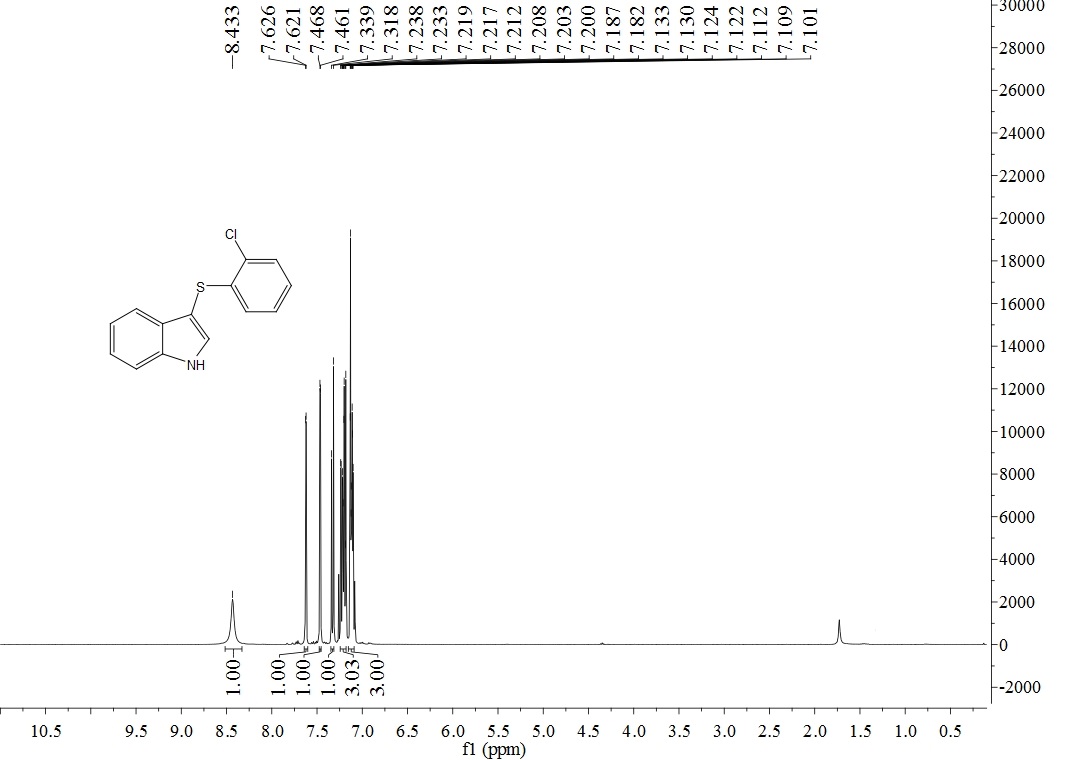
**

**
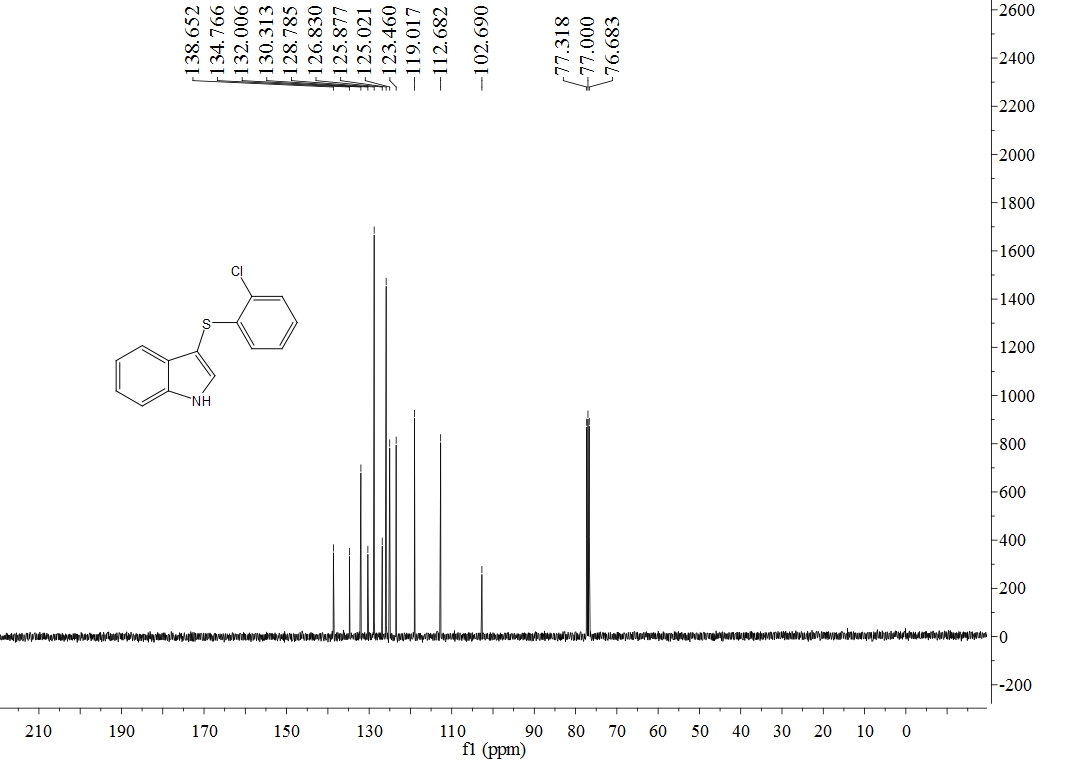
**

**3j**

**
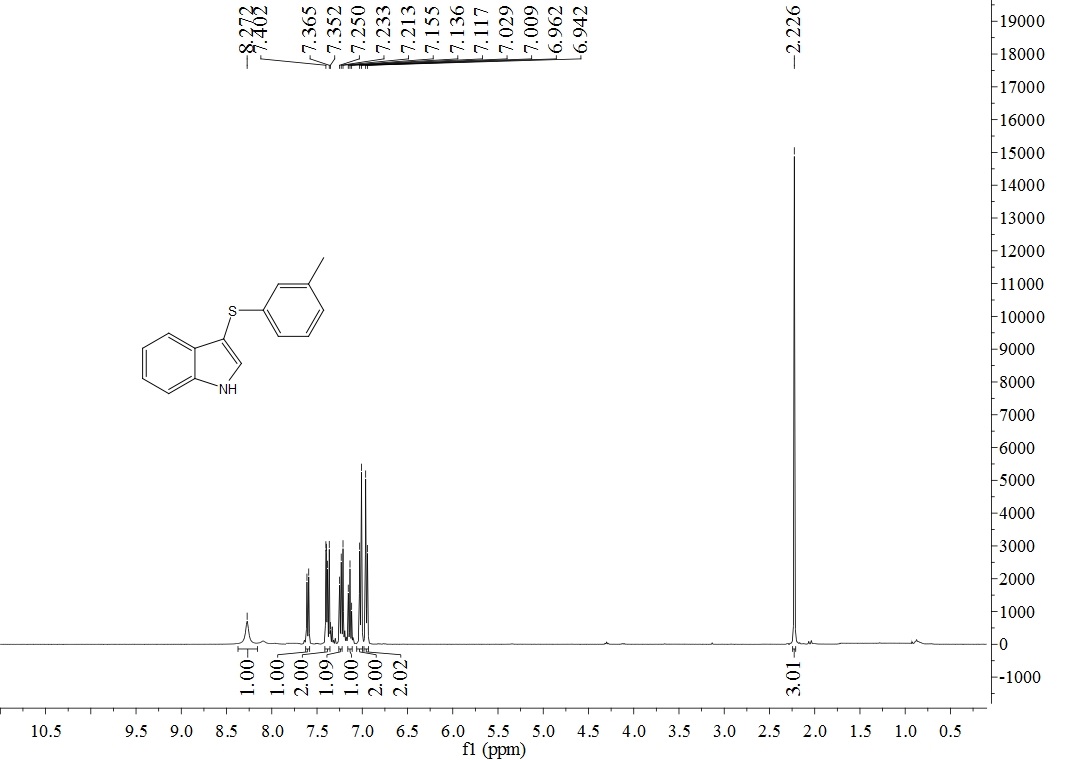
**

**
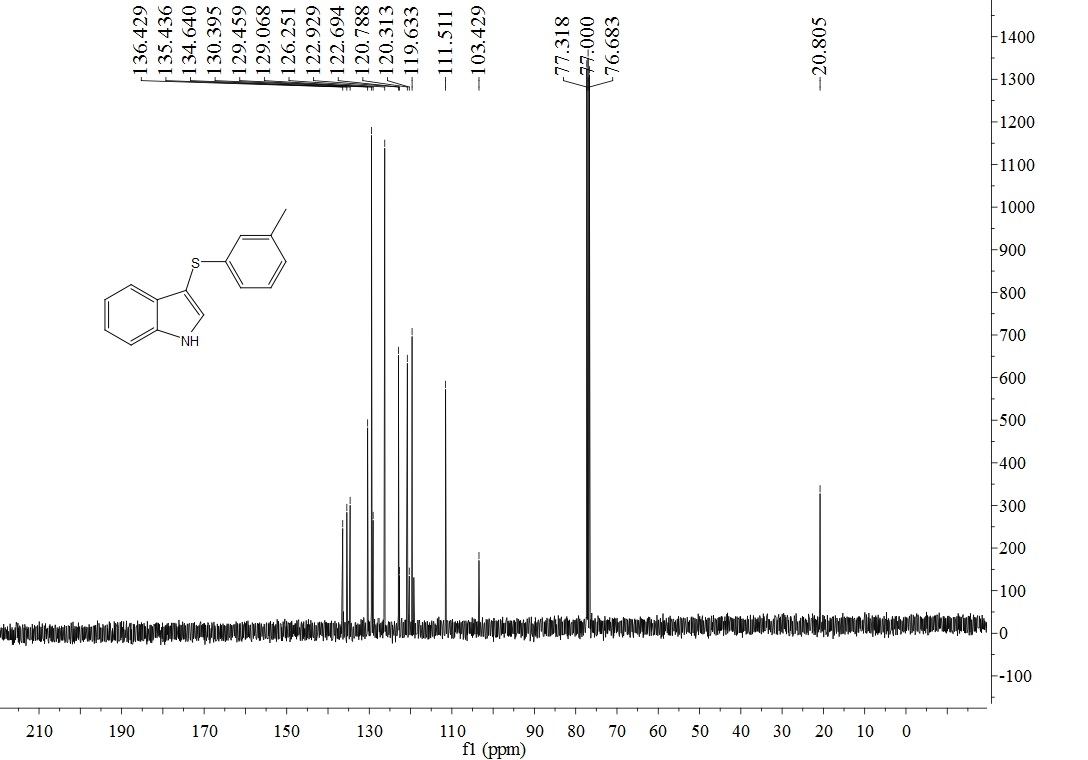
**

**3k**

**
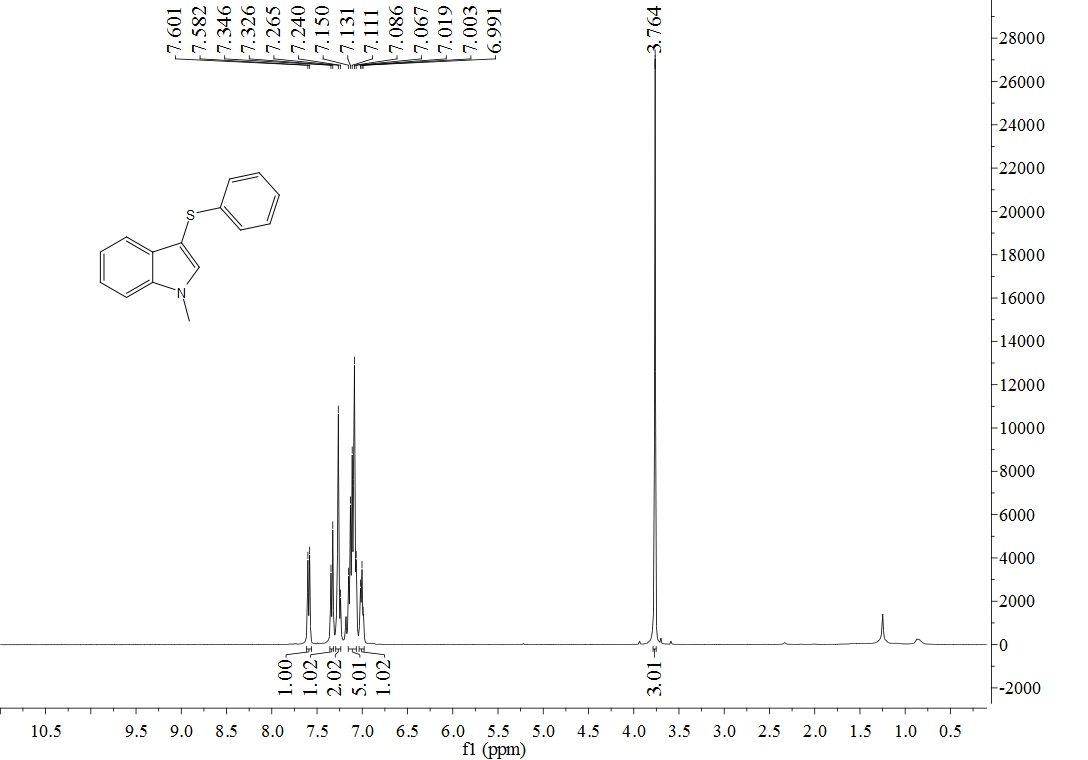
**

**
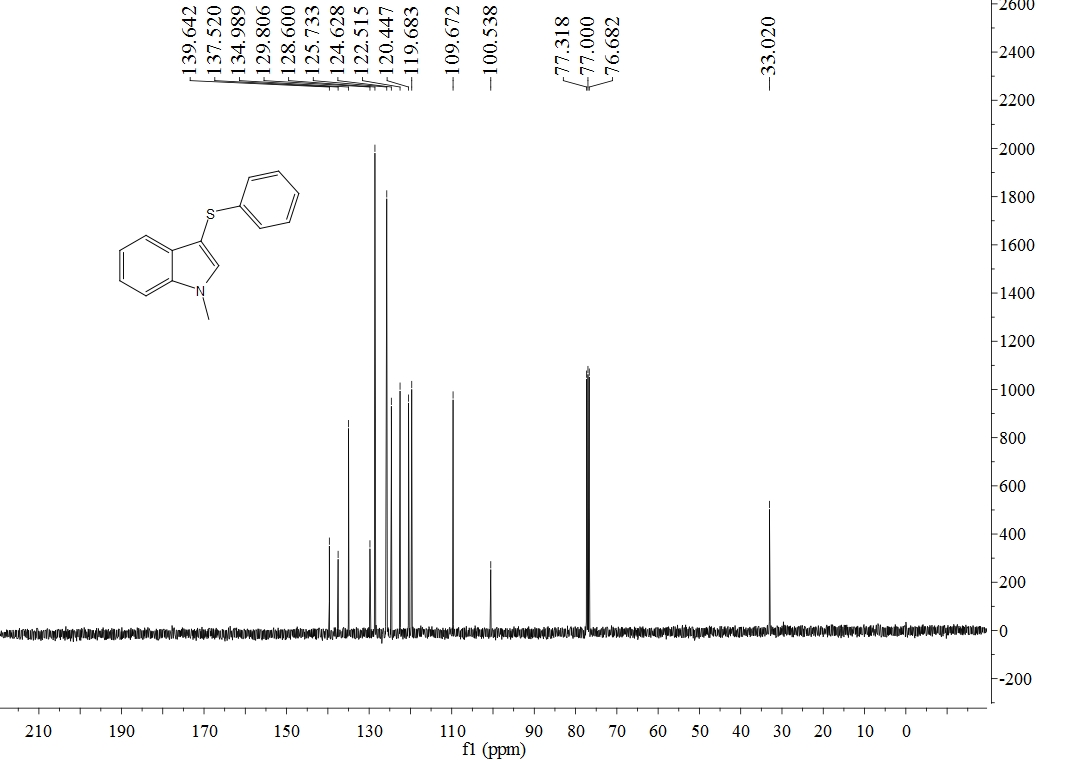
**

**3l**

**
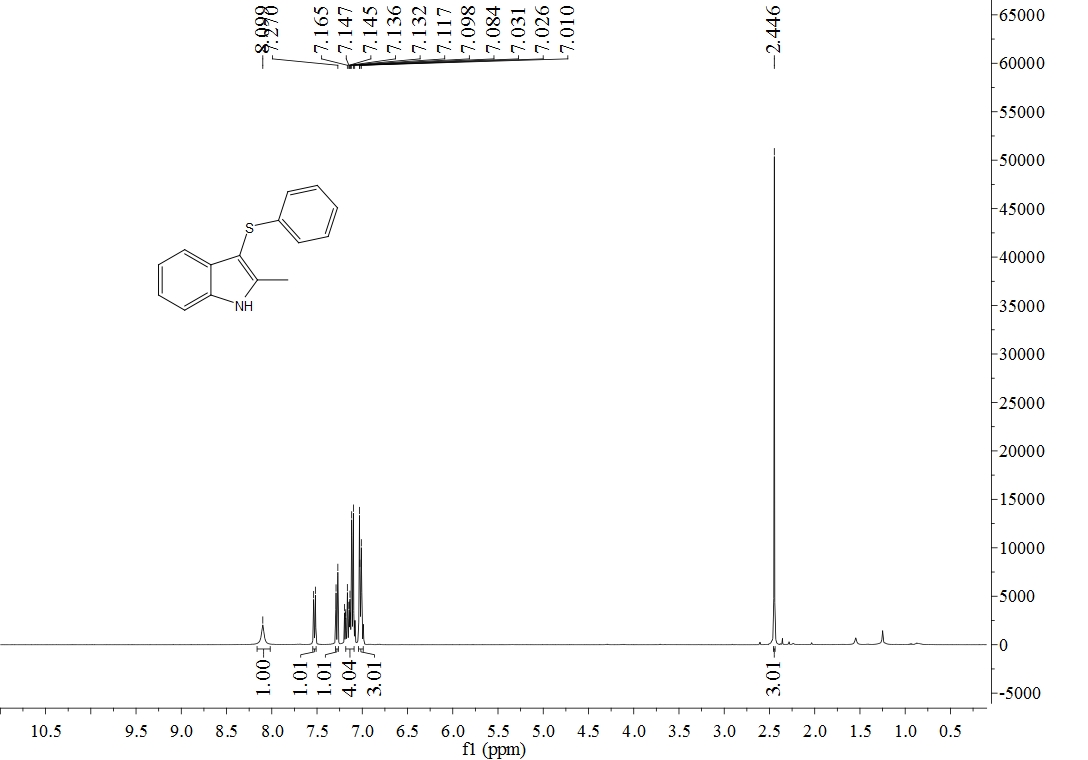
**

**
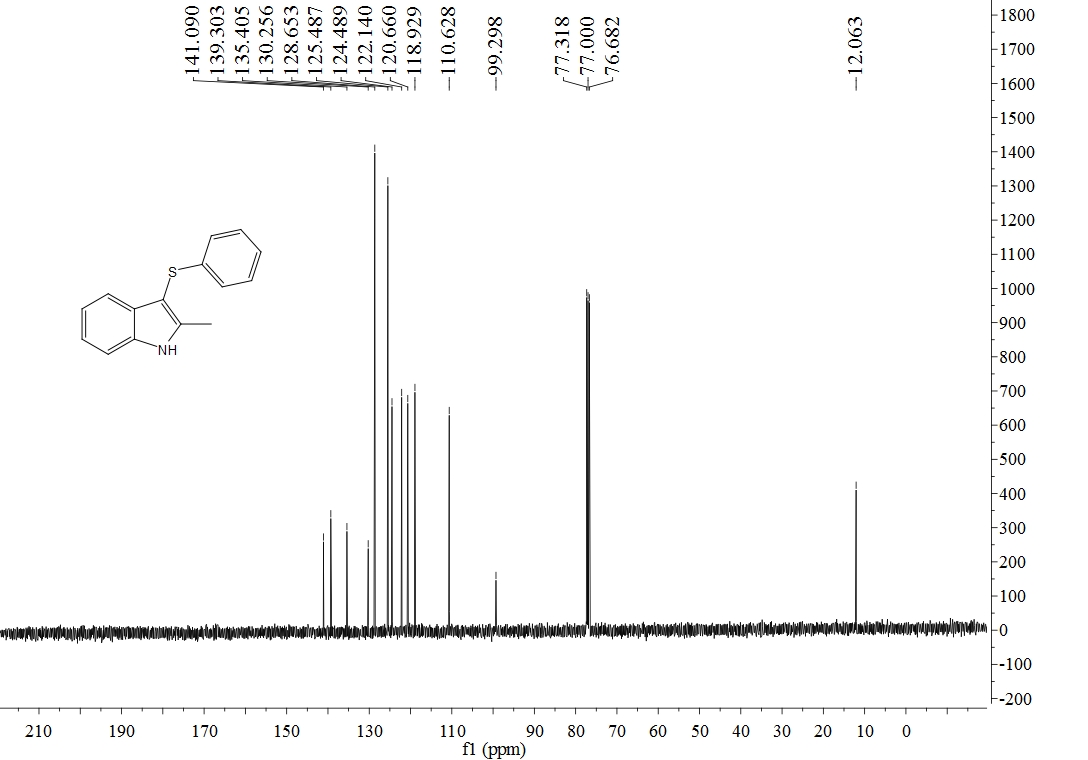
**

**3m**

**
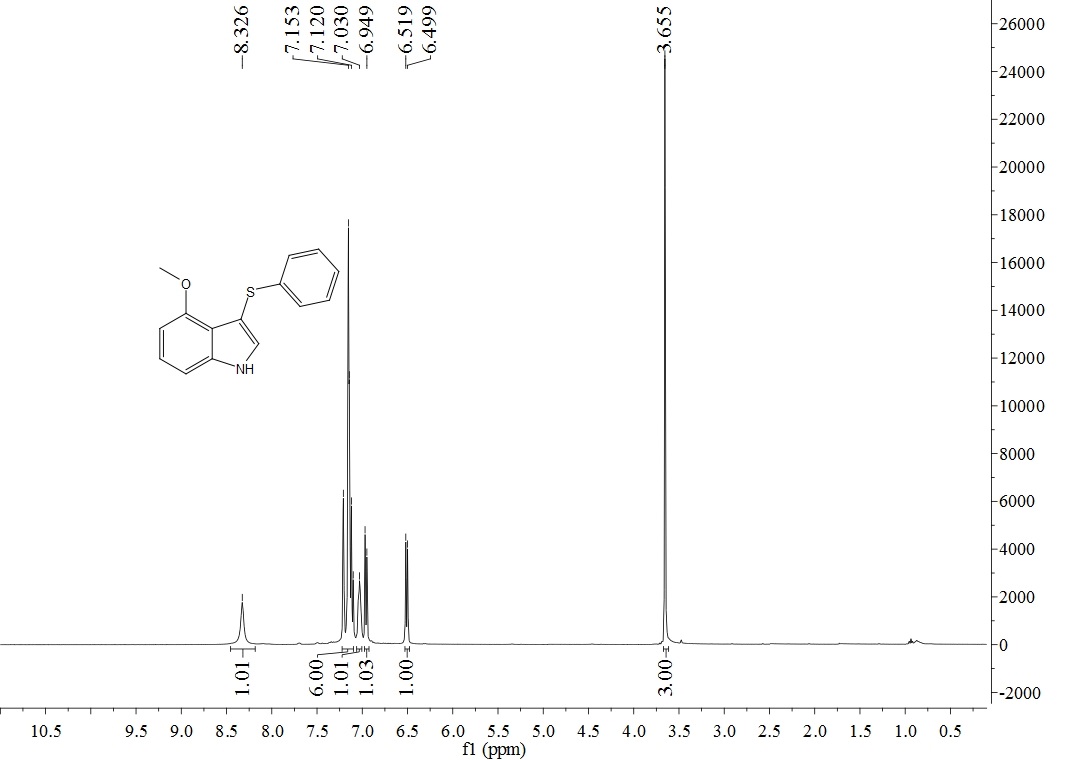
**

**
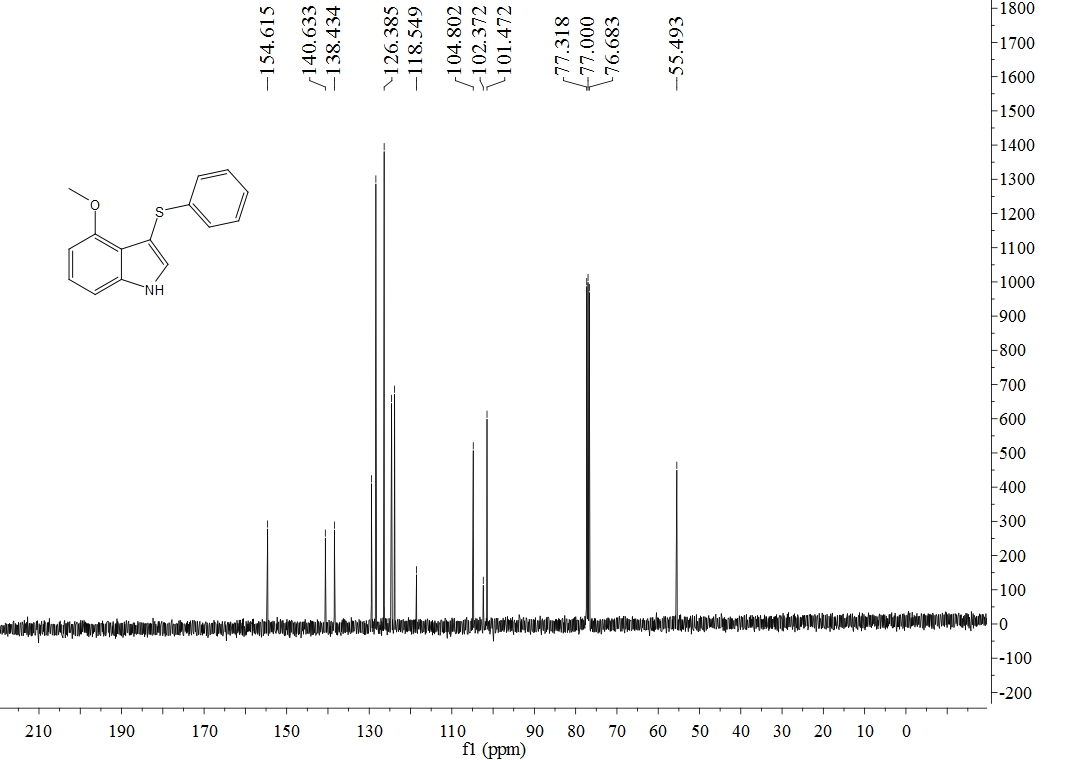
**

**3n**

**
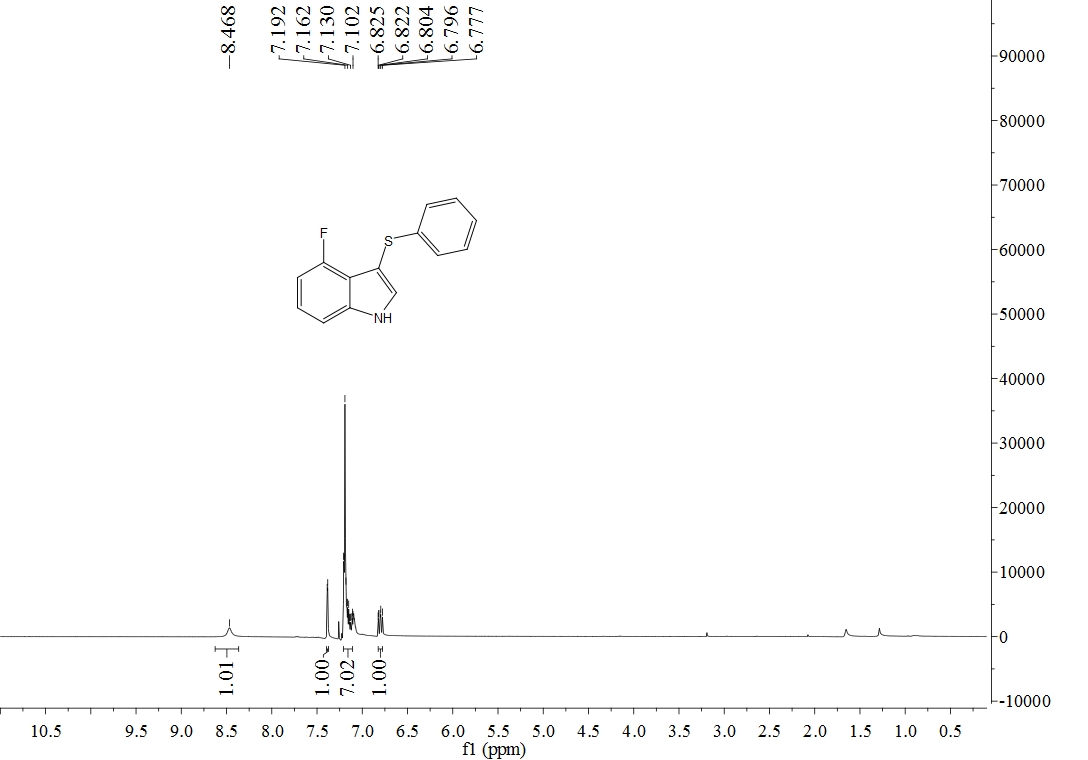
**

**
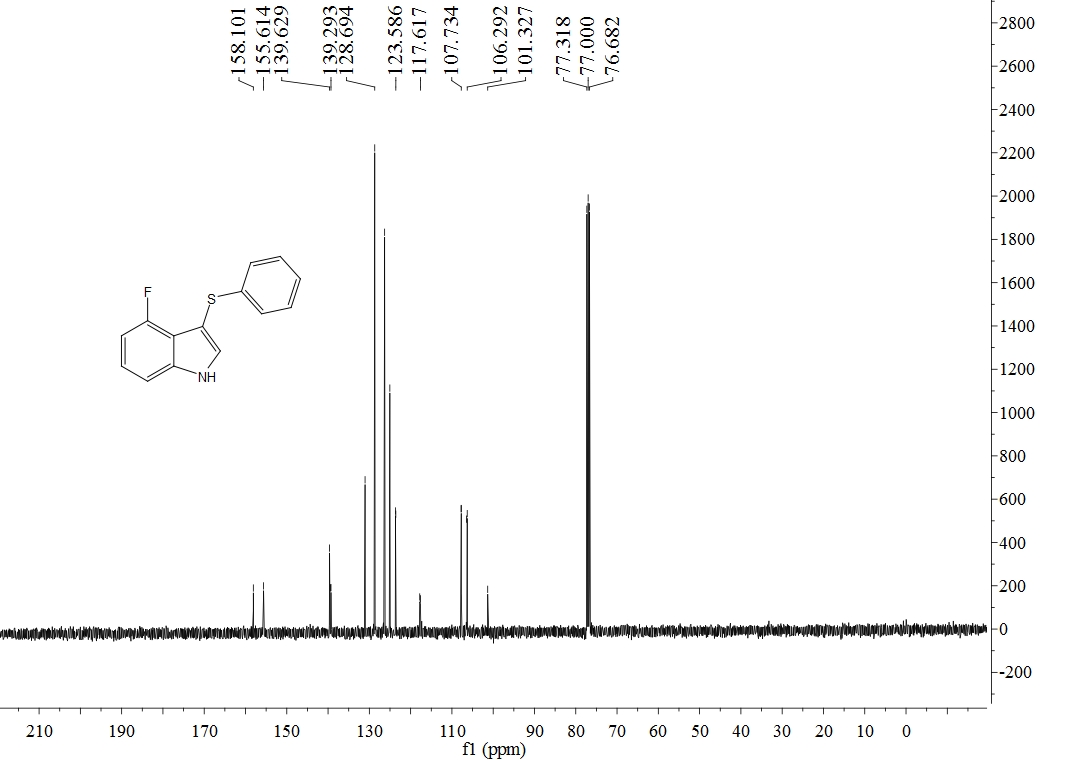
**

**3o**

**
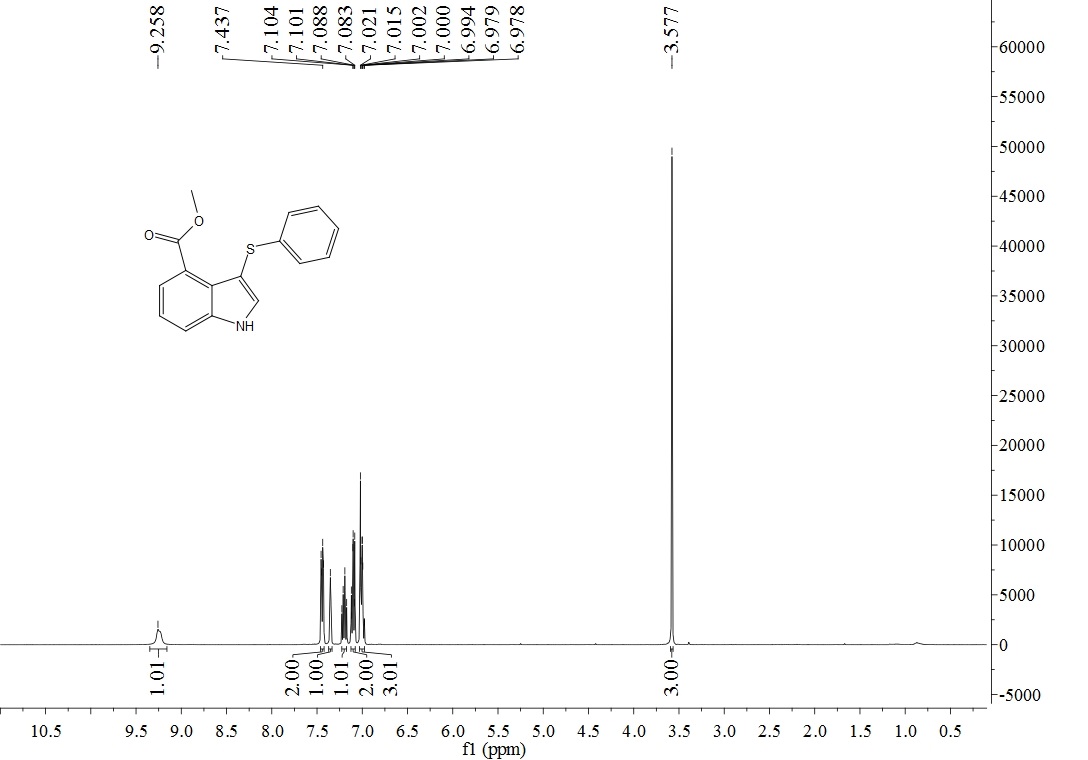
**

**
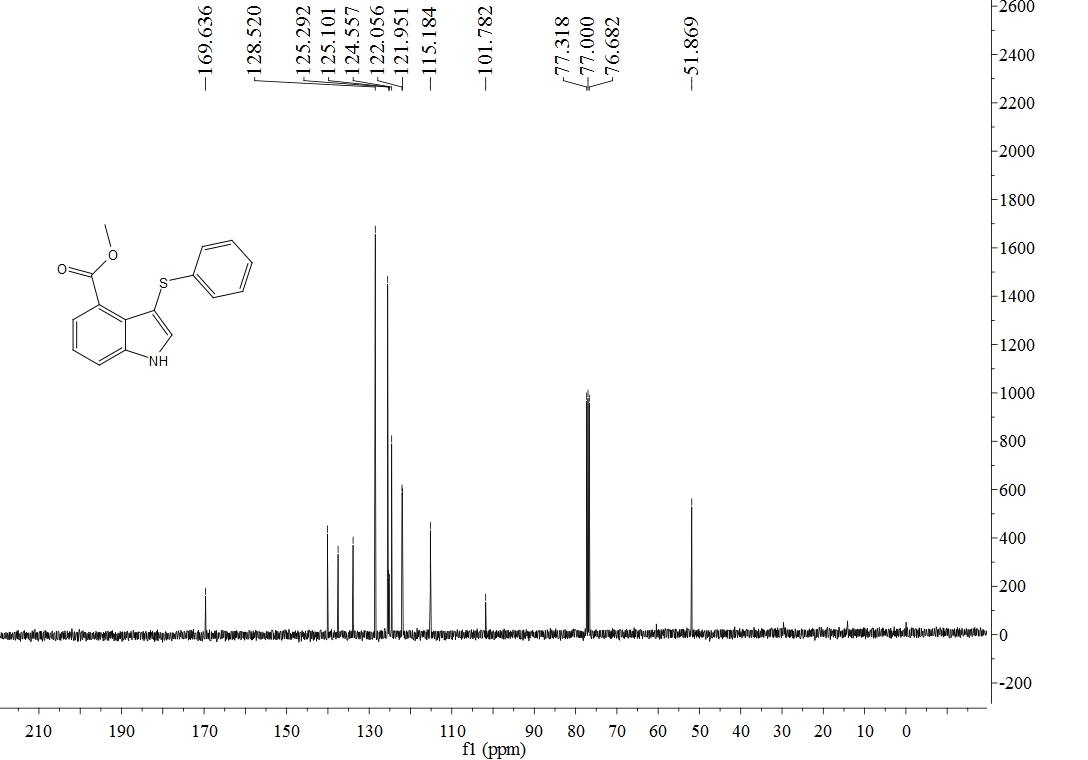
**

**3p**

**
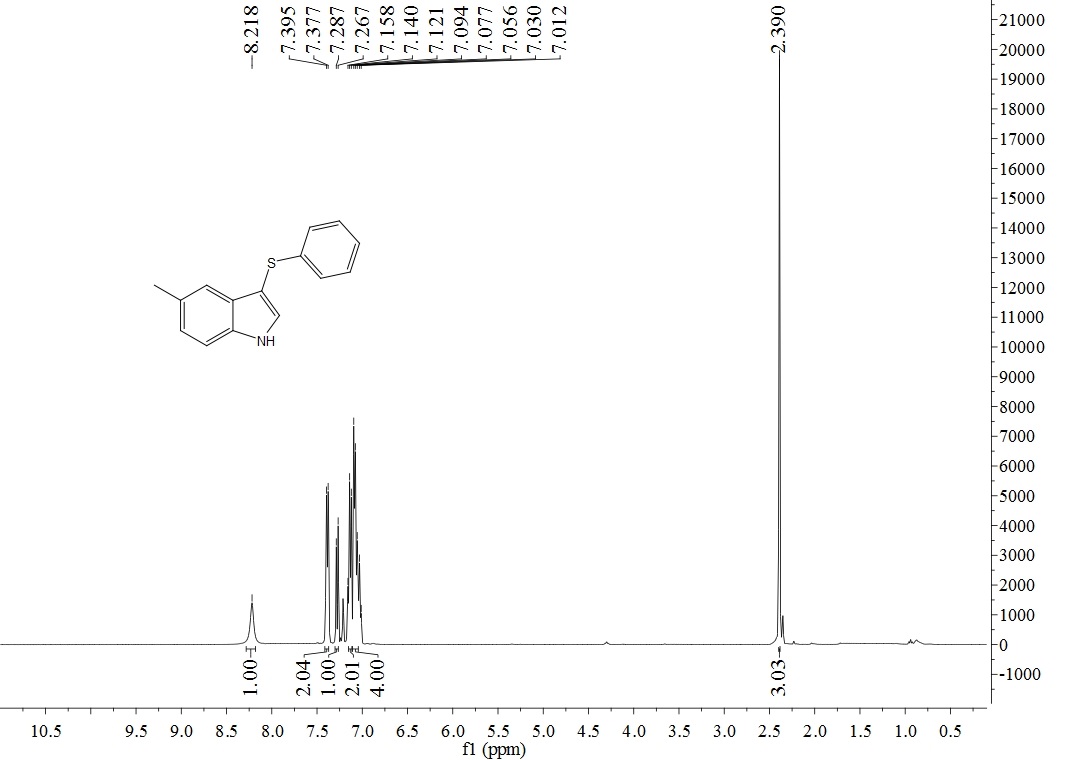
**

**
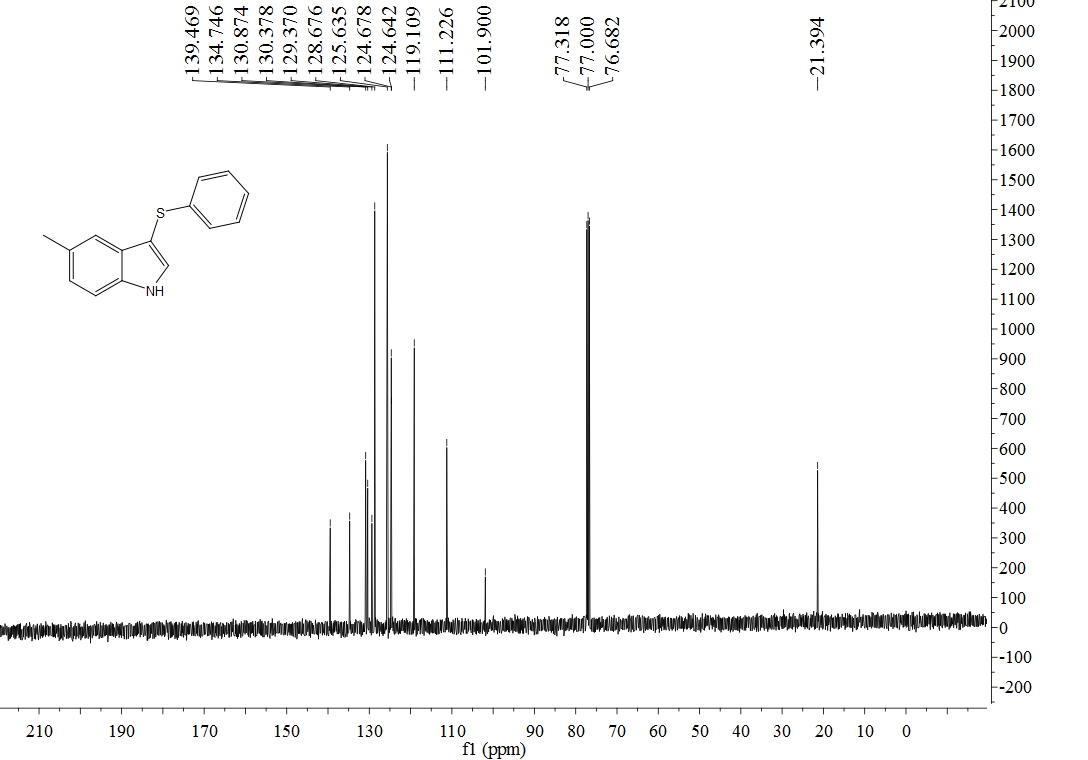
**

**3q**

**
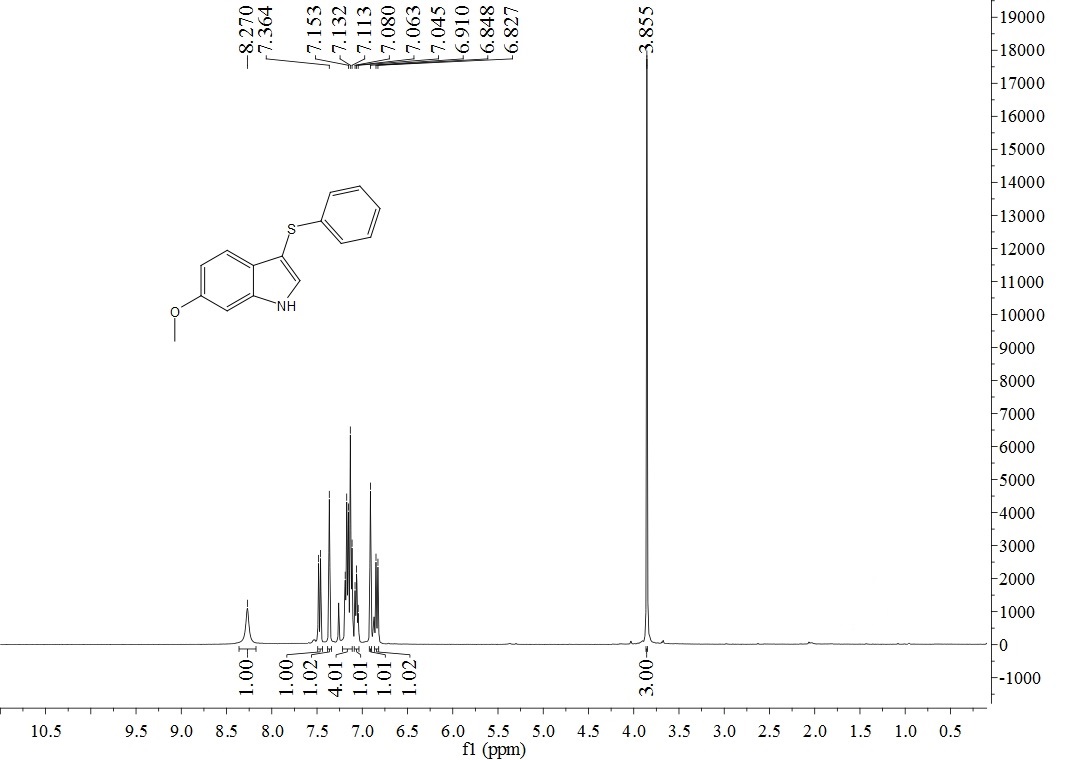
**

**
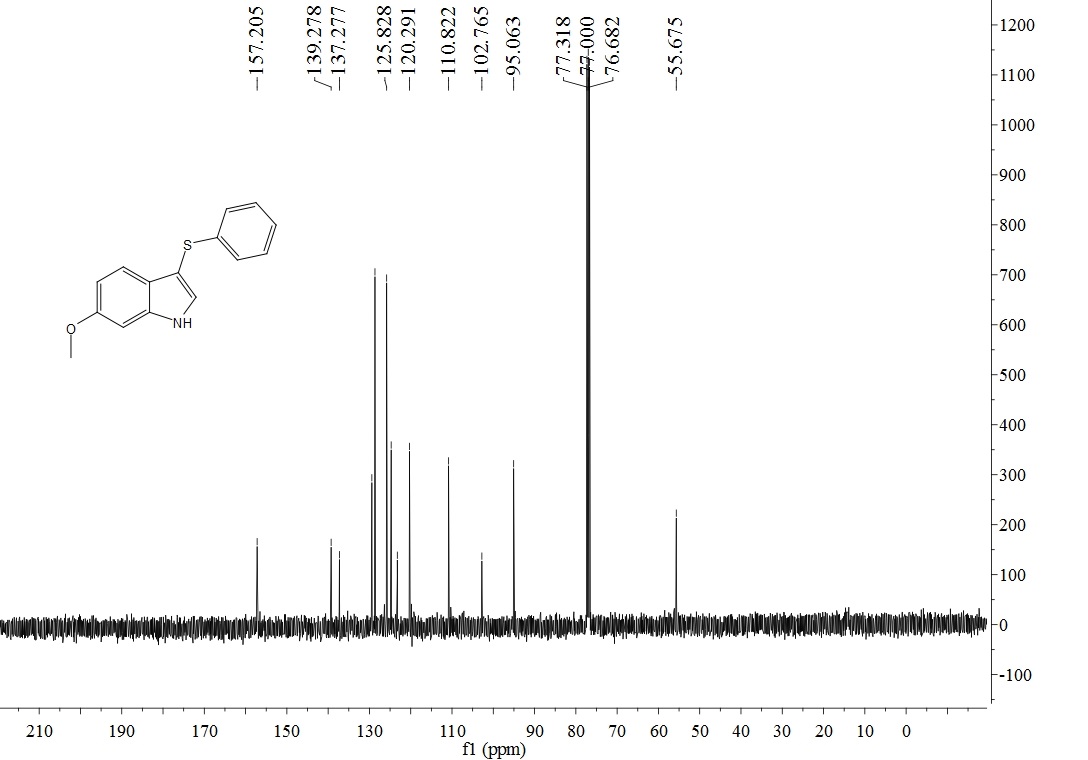
**

**3r**

**
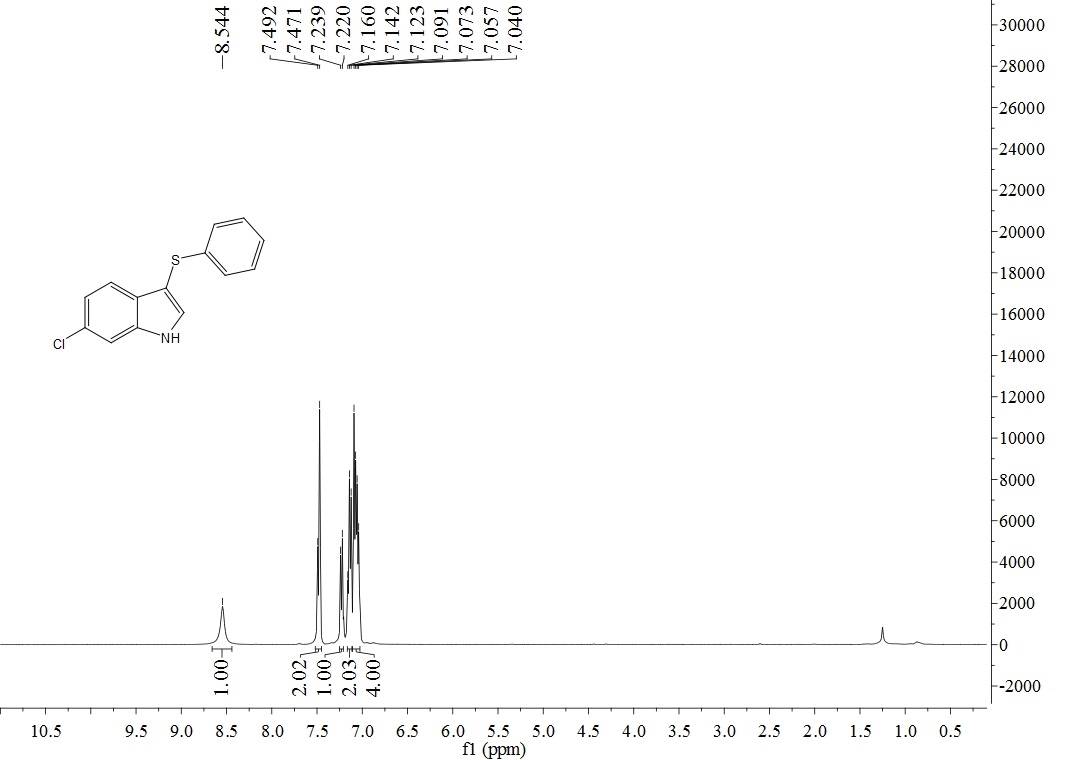
**

**
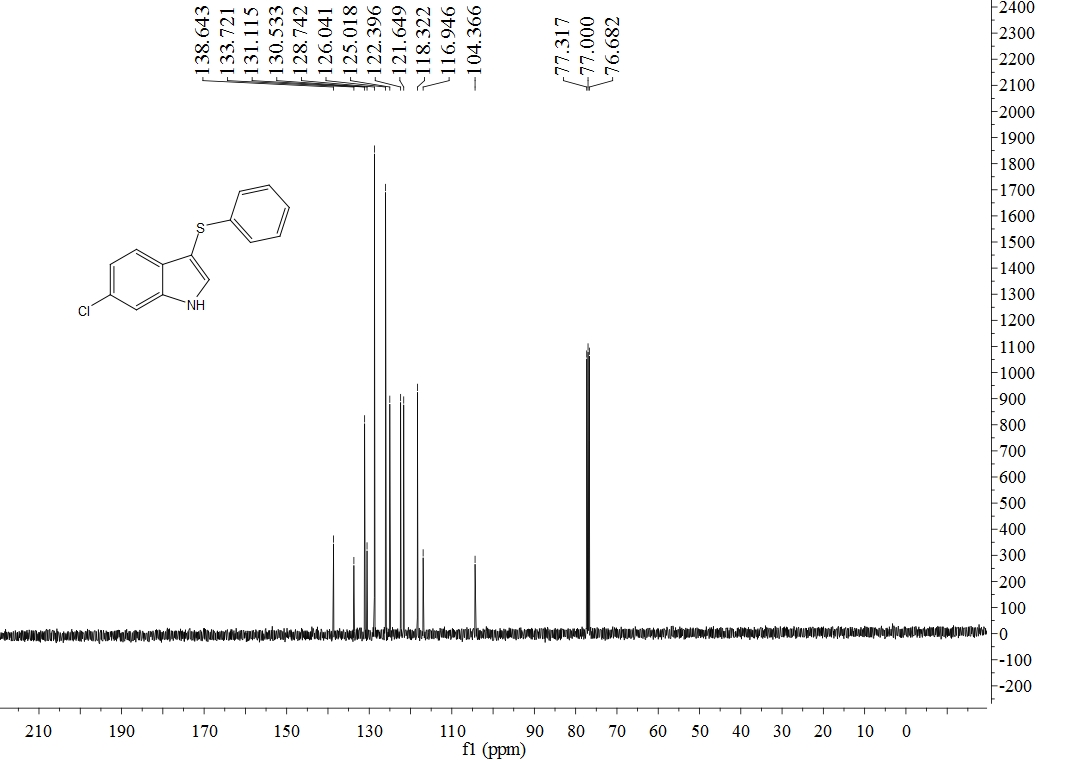
**

**3s**

**
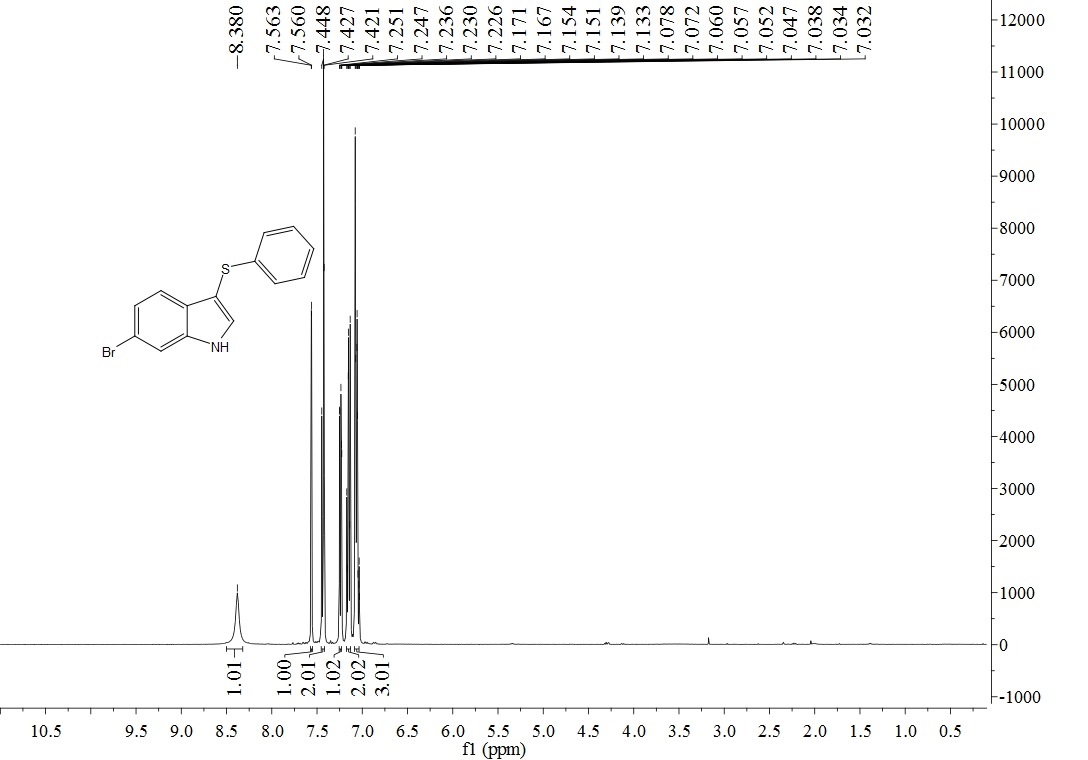
**

**
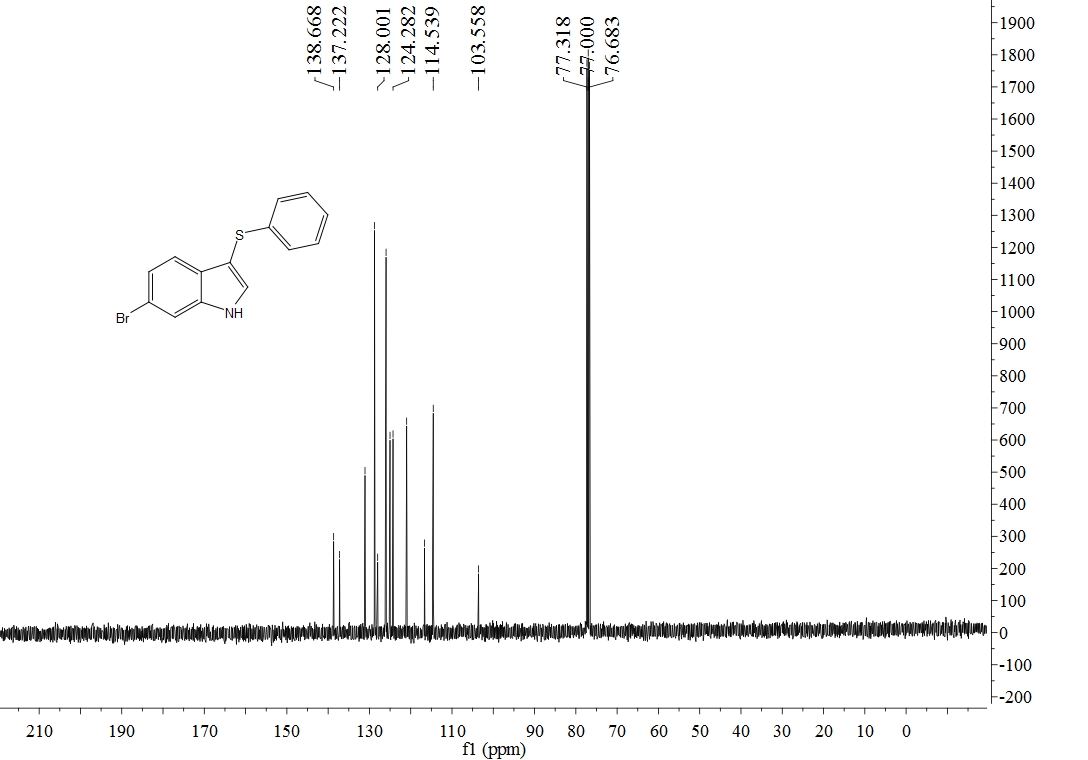
**

**3t**

**
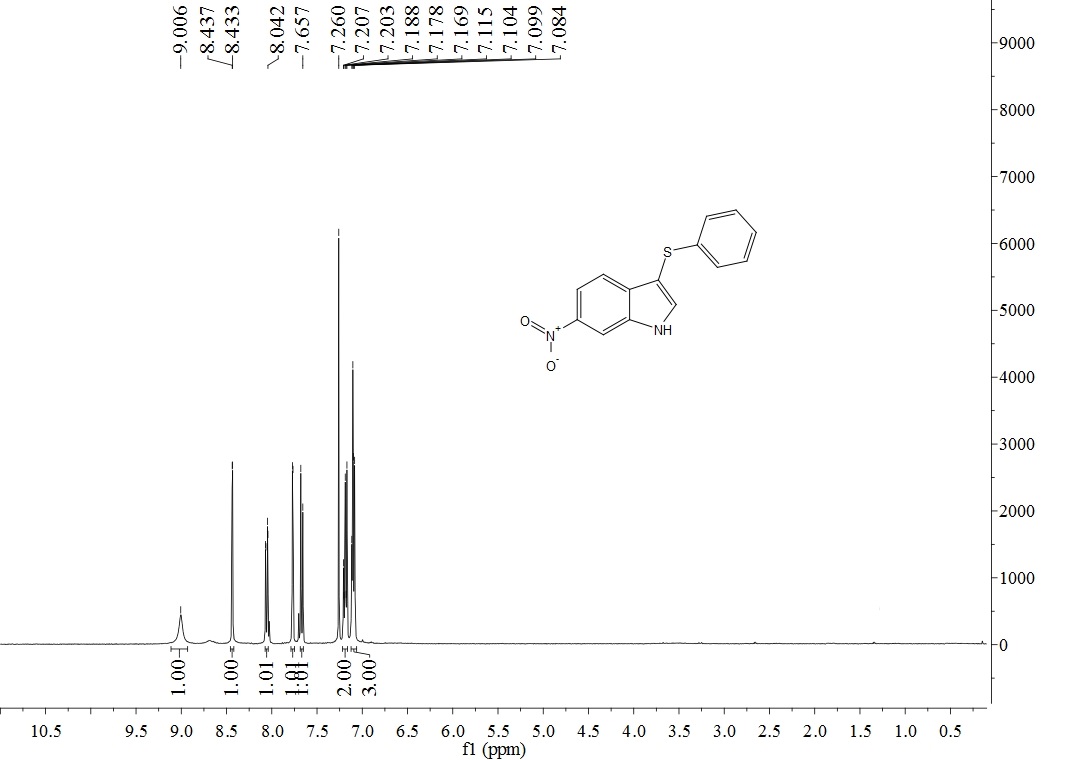
**

**
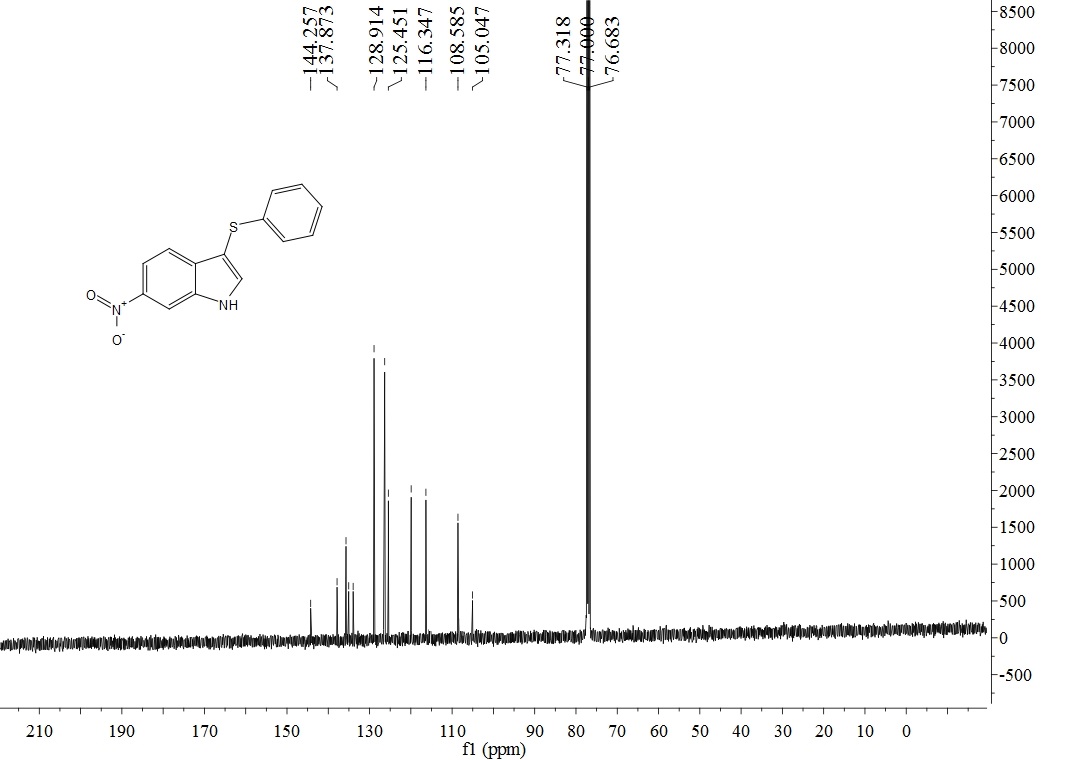
**

**3u**

**
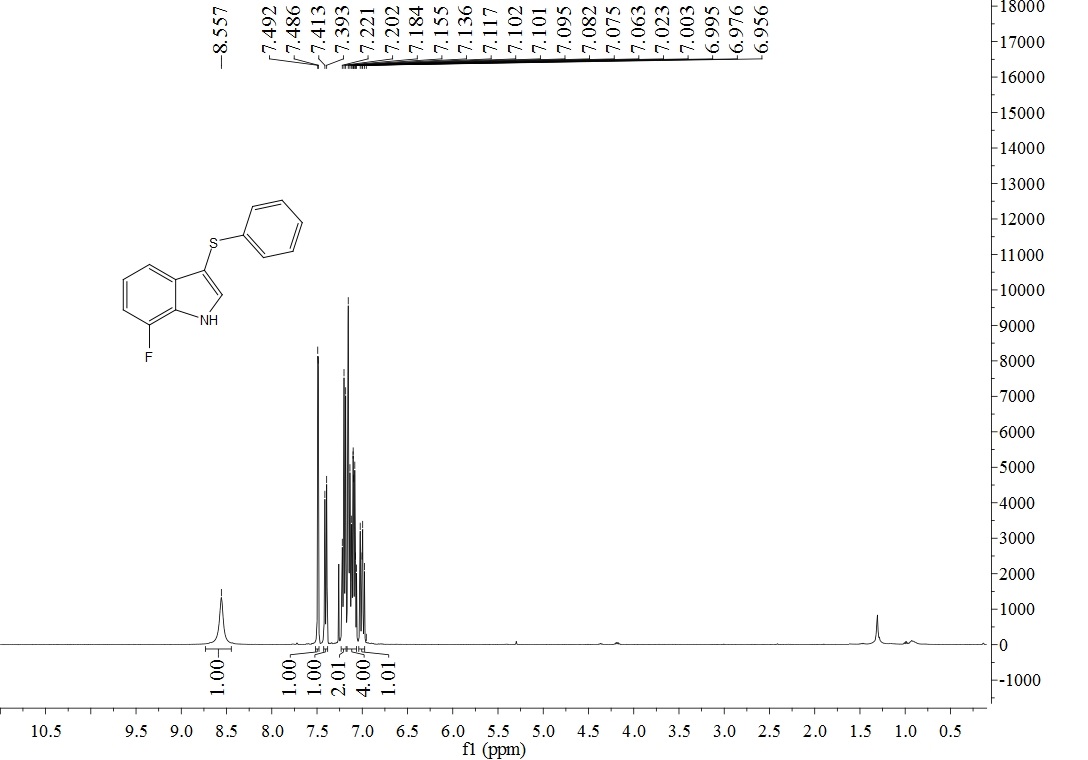
**

**
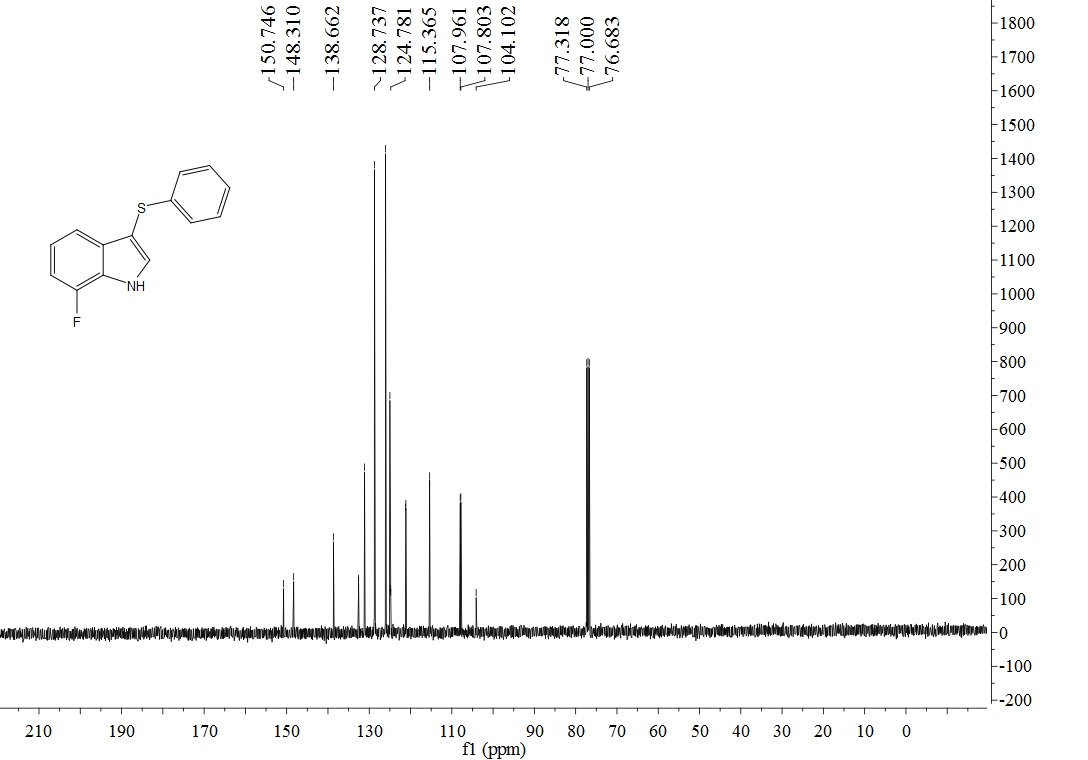
**

**3v**

**
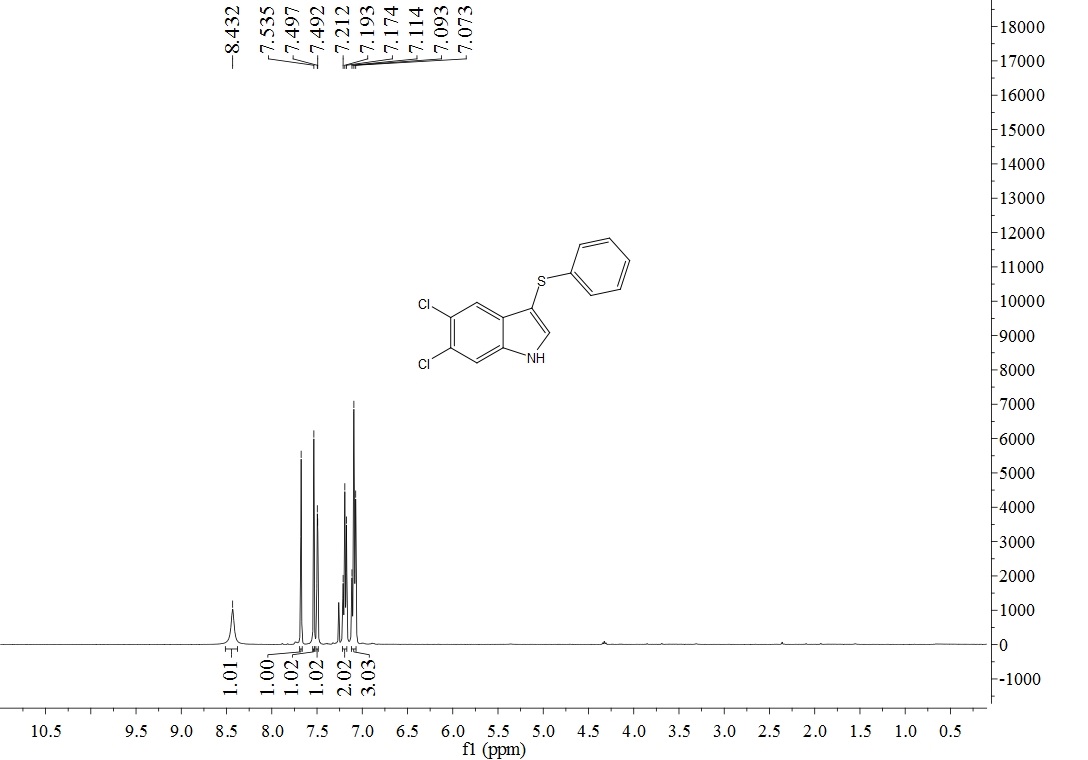
**

**
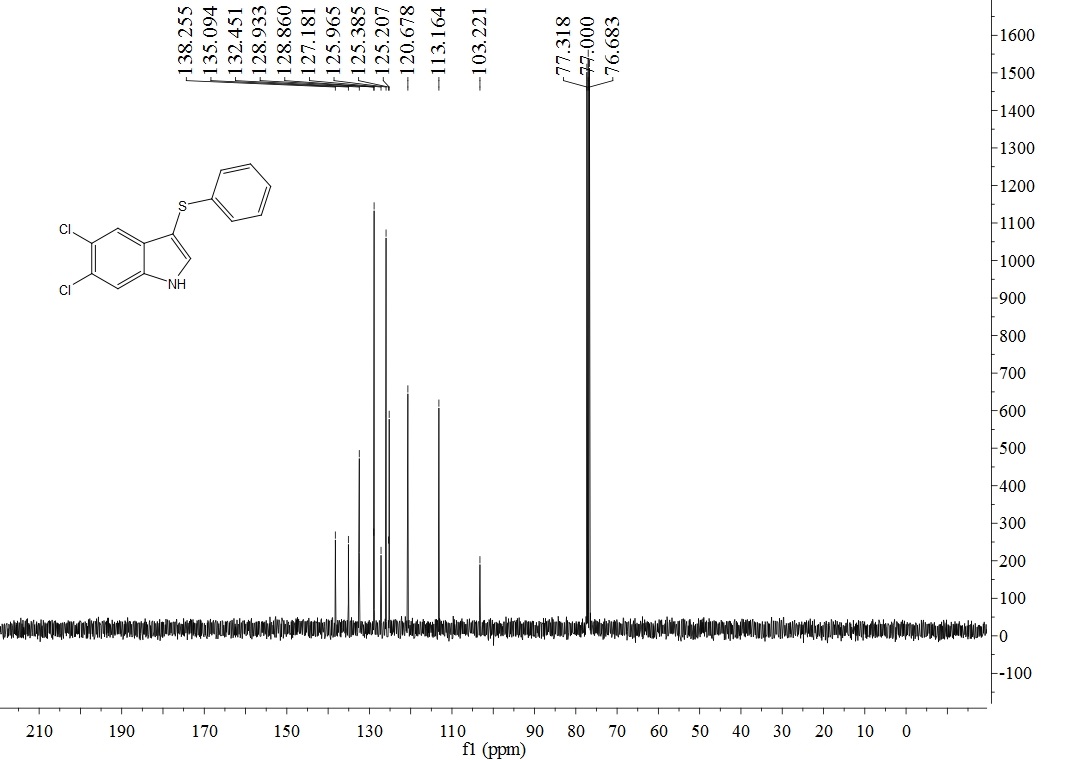
**

**3w**

**
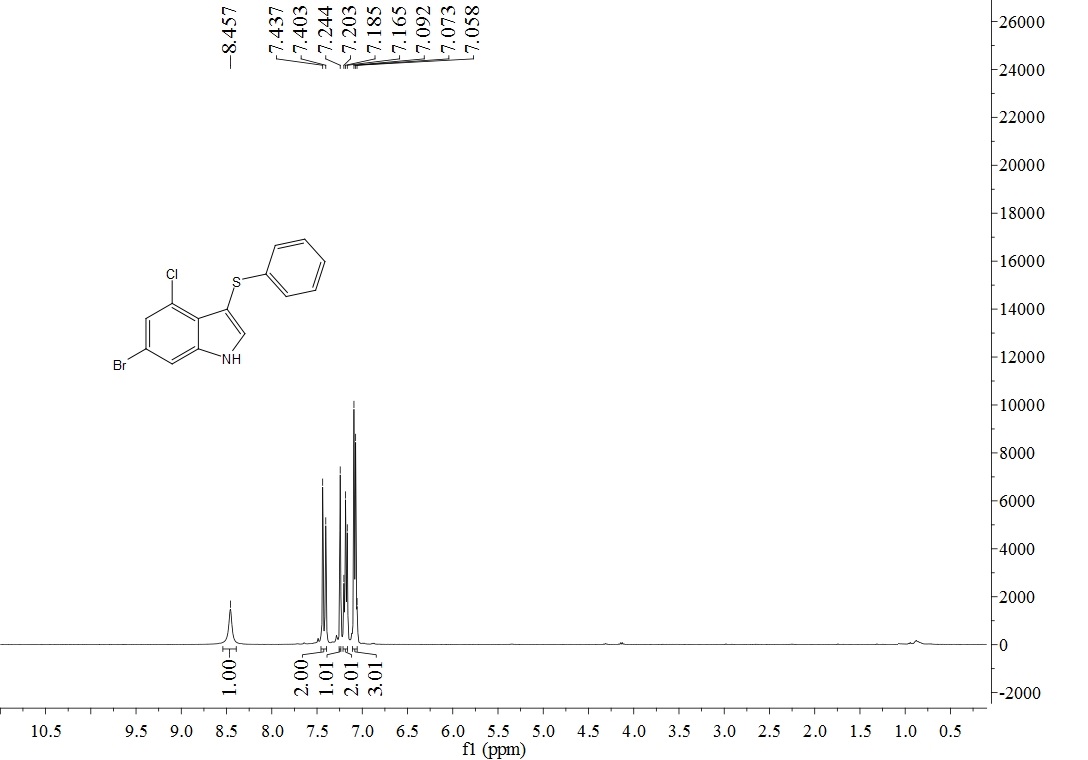
**

**
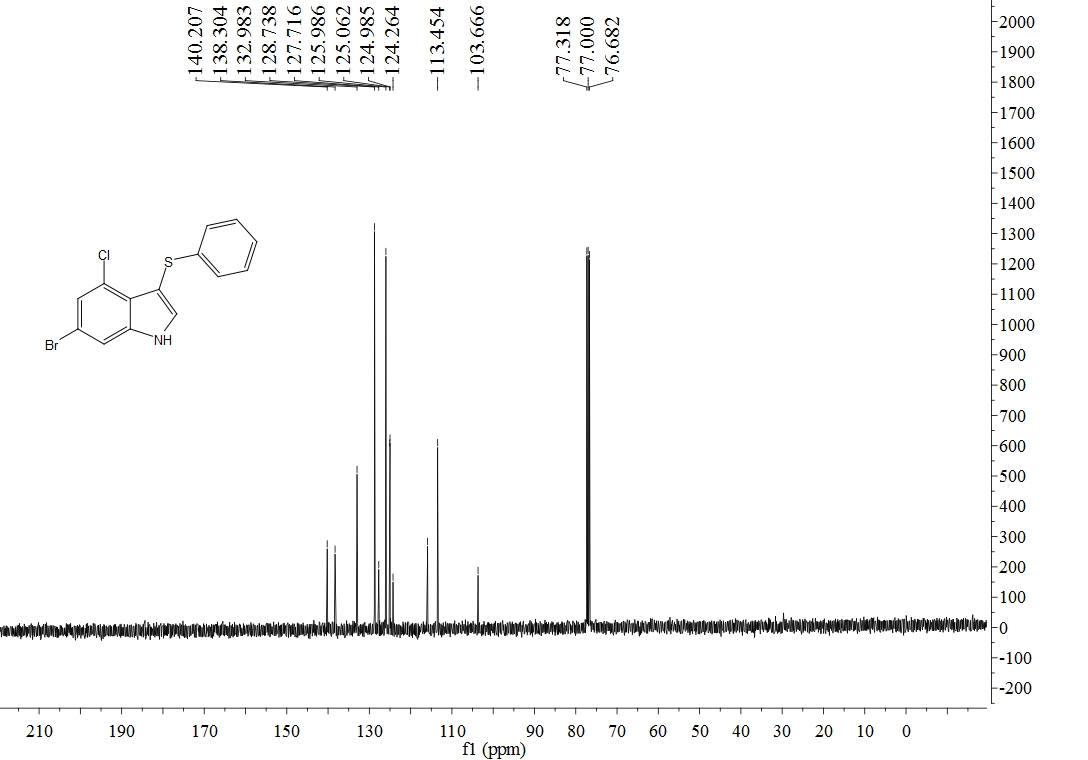
**
